# Exploring the CD38-Associated Surfaceome *via* Nanobody-Targeted TurboID

**DOI:** 10.1016/j.mcpro.2026.101623

**Published:** 2026-07-14

**Authors:** Kangze Feng, Ying Wei, Shangcheng Yu, Wenxuan Hou, Meng Han, Yuling Chen, Yuxiang Tang, Haiteng Deng

**Affiliations:** 1 MOE Key Laboratory of Bioinformatics, Center for Synthetic and Systematic Biology, State Key Laboratory of Complex, Severe, and Rare Diseases, School of Life Sciences, Tsinghua University, Beijing, China; 2 Zhejiang Key Laboratory of Multiomics and Molecular Enzymology, Yangtze Delta Region Institute of Tsinghua University, Jiaxing, China

**Keywords:** proximity labeling, nanobody, CD38, surfaceome nanoscale organizations, cell migration

## Abstract

Cluster of differentiation (CD38) is a cell-surface glycoprotein whose molecular functions are closely linked to its surrounding protein environment. To date, omics-level understanding of CD38-associated surface proteins remains lacking. To investigate the CD38-proximal surfaceome in live adherent cell culture, we developed nanobody-targeted TurboID (NBID), a proximity labeling approach using protein of interest–specific nanobody-TurboID chimeras, and applied this approach to characterize CD38-proximal proteome on the cell surface. The approach was validated using epidermal growth factor receptor-targeting NTC construct, which recovered multiple known epidermal growth factor receptor-associated proteins. CD38-specific NTC was applied to A549 lung cancer cells and Tohoku Hospital Pediatrics-1 monocytic leukemia cells, and enriched multiple proteins involved in adhesion, ECM organization, and lipid raft-associated membrane domains. In A549 cells, proteins related to Wnt signaling were additionally enriched. NAD^+^ treatment did not significantly alter the CD38-proximal surfaceome in A549 cells. Total internal reflection fluorescence microscopy further demonstrated coclustering of several enriched candidates with CD38 on membrane protrusions. Functional assays showed that CD38 contributes to transendothelial migration of A549 cells. By combining NBID with stable isotope labeling by amino acids in cell culture, we further identified CD38-associated cadherin adhesion complexes involving N-cadherin and desmoglein-2 at the tumor–endothelial interface. Together, these results establish NBID as a robust platform for investigating surface protein environments and intercellular protein neighborhoods under native conditions. Furthermore, our findings reveal a CD38-associated membrane adhesion network and provide new insight into the role of CD38 in tumor cell migration and tumor–endothelial interactions.

## Highlights

• Nanobody-TurboID chimeras enables identification of surface protein organizations.
• We report the surface proximal proteome of CD38 in various cell models.
• CD38 positively regulates tumor migration in A549 cells.
• CD38 is associated with tumor-endothelial cadherin adhesion complexes.

## Graphical Abstract

**Figure:**
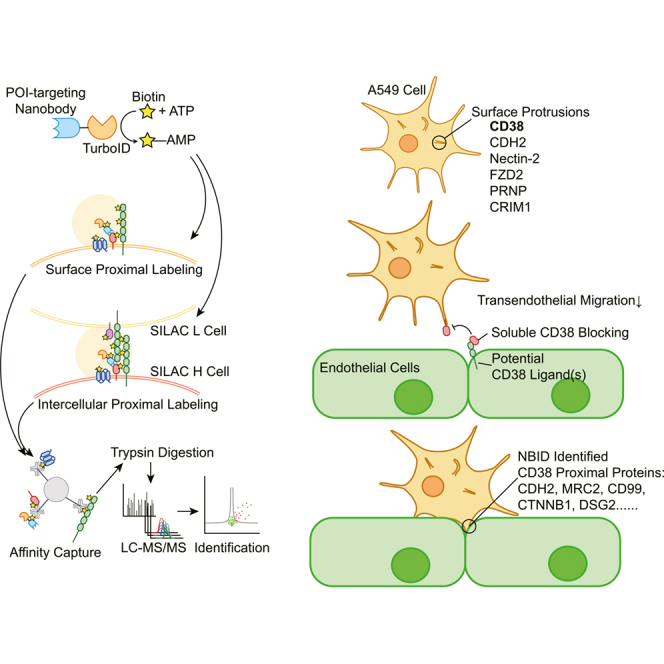

## In Brief

We developed nanobody-targeted TurboID (NBID), a proximity labeling approach for mapping protein environments at the cell surface under cultured conditions. Application of NBID generated a comprehensive view of the CD38-proximal surfaceome across different cell types and uncovered CD38-associated cadherin adhesion complexes involved in tumor–endothelial interactions. NBID provides a broadly applicable strategy for studying membrane protein organization and intercellular protein networks.

Cellular behavior is tightly regulated by protein interactions at the cell surface, which control processes such as signal transduction, adhesion, migration, and extracellular matrix (ECM) remodeling (1, 2, 3). These functions are increasingly recognized to depend not only on individual proteins but also on their spatial organization within dynamic membrane-associated networks. Understanding how surface proteins are organized into these nanoscale assemblies is therefore essential for elucidating the molecular mechanisms underlying both physiological and pathological cellular processes. Among the membrane proteins implicated in these processes, cluster of differentiation (CD38) has emerged as a multifunctional ectoenzyme and receptor involved in immunity, metabolism, and cancer progression (4).

CD38 is a multifunctional transmembrane glycoprotein initially identified on immune cells and subsequently found in diverse cell types, including pancreatic β-cells, neurons, and solid tumors (4, 5, 6). Two membrane orientations of CD38 have been described. In the canonical type II orientation, the catalytic domain is exposed extracellularly, whereas the less abundant type III orientation positions the catalytic domain toward the cytoplasm (7). CD38 catalyzes the hydrolysis of NAD+ and the generation of signaling molecules such as cyclic ADP-ribose, linking it to calcium signaling and cellular metabolism (7, 8). Beyond its enzymatic activity, CD38 also functions as a receptor and adhesion molecule through interaction with proteins such as PECAM-1 (CD31), thereby contributing to immune cell activation, adhesion, and migration (9). Notably, the receptor functions of type II CD38 are thought to depend on lateral associations with neighboring membrane proteins, as its short cytoplasmic tail lacks canonical signaling motifs (10, 11). Supporting this notion, CD38 has been identified as part of a lipid raft-associated signaling complex containing CD19 and CD81 in B cells, suggesting that membrane-associated protein networks play a central role in mediating CD38 receptor function (12). CD38 has been extensively studied in hematological malignancies, where it serves as both a disease-associated molecule and a therapeutic target (13), and emerging evidence suggests that it also promotes progression and migration in several solid tumors (4). In lung cancer cells, CD38-mediated depletion of intracellular NAD^+^ has been reported to promote epithelial–mesenchymal transition (EMT) (14). However, most studies in cancer have focused on the enzymatic functions of CD38, whereas the membrane-associated protein networks that may mediate its receptor functions in solid tumors remain poorly characterized.

Systematic characterization of membrane-associated protein networks has become increasingly feasible with the development of spatial proteomics approaches. Among these, proximity labeling (PL) methods enable *in situ* mapping of protein neighborhoods by covalently tagging proteins located within a defined radius of a protein of interest (POI), followed by affinity enrichment and mass spectrometry-based identification. Enzyme-based PL approaches such as BioID (15), TurboID (16), and APEX (17) have been widely applied to characterize both intracellular protein complexes and cell-surface microenvironments. However, canonical PL approaches rely on genetic fusion of the labeling enzyme to the POI, which may perturb native protein organization and limit their applicability in systems where genetic manipulation is undesirable.

To overcome the requirement for genetic fusion, various antibody-directed PL strategies have been developed, including EMARS, BAR, ProtA-TurboID, LUX-MS, and IPL (18, 19, 20, 21, 22). These approaches enable profiling of endogenous proteins in their native cellular context and have expanded the applicability of PL beyond genetically tractable systems. However, studying membrane-associated lateral and intercellular protein networks under native adherent culture conditions can still present practical challenges for antibody-based workflows, particularly when reagent requirements increase substantially with culture scale or when additional conjugation steps are required. To address these limitations, we explored the use of nanobodies, small (∼15 kDa) and stable antibody fragments derived from camelid heavy-chain antibodies that can be readily produced recombinantly (23). Based on these properties, we developed nanobody-targeted TurboID (NBID), a PL strategy employing purified POI-specific nanobody–TurboID chimeras (NTCs) to directly target endogenous surface proteins in live-cell cultures without genetic manipulation. This approach was developed to enable characterization of the membrane-associated protein network of CD38 under native culture conditions.

In this study, we first validated NBID using an epidermal growth factor receptor (EGFR)-targeting nanobody and recovered multiple known EGFR-associated proteins. We then applied NBID using the previously characterized CD38-specific nanobody MU1053 (24) to investigate the membrane-associated protein network of endogenous CD38 in A549 lung cancer cells and Tohoku Hospital Pediatrics-1 (THP-1) monocytic leukemia cells. Functional experiments implicated CD38 in tumor transendothelial migration (TEM). Using a stable isotope labeling by amino acids in cell culture (SILAC)-labeled tumor–endothelial coculture system, NBID further revealed a CD38-associated intercellular cadherin adhesion network involving N-cadherin (CDH2) and desmoglein-2. Together, these findings establish NBID as a platform for studying endogenous cell-surface protein organization and provide a framework for understanding how CD38-associated membrane protein networks contribute to tumor cell behavior.

## Experimental Procedures

### Plasmid Constructs

Nanobody MU1053, nanobody 7D12, and TurboID sequences were synthesized by Xianghong Biological. A Strep-tag II sequence was added to the C terminus by PCR. NBID fusion constructs were generated by overlap PCR and cloned into the pET-28a(+)-small ubiquitin-like modifier (SUMO) vector using the Seamless Cloning Kit (Beyotime).

Full-length cDNAs of CD38, CDH2, CRIM1, FZD2, nectin-2 (NECTIN2), and PRNP were amplified from A549 cell cDNA. CD38 was cloned into the pLVX-IRES-zsGreen1 vector, and all six genes were also cloned into pCDNA3.1(−). For fluorescent protein (FP) fusion constructs, mCherry or EGFP was fused to either the N terminus or C terminus of the mature protein, depending on the construct design.

### Cell Culture, SILAC Labeling, and Coculture

Human non–small cell lung cancer A549, human monocytic leukemia THP-1, and human embryonic kidney HEK293T cells were cultured in RPMI-1640, RPMI-1640, and DMEM medium (all from Wisent), respectively, supplemented with 10% fetal bovine serum (FBS; PAN) and 1% penicillin streptomycin (P/S; Wisent). SV40T transfected human umbilical vein endothelial cells (HUVEC-T1) were cultured in DMEM/F12 medium supplemented with 15% FBS and 1% P/S.

For SILAC, A549 cells were cultured for at least 10 doublings in SILAC RPMI-1640 medium (Thermo Fisher Scientific) supplemented with 10% dialyzed FBS (ScienProCell), 1% P/S, ^13^C_6_-lysine and ^13^C_6_^15^N_4_-arginine (Cambridge Isotope Laboratory).

For NBID labeling in monocultures, A549 and HeLa cells were grown to ∼80% confluence in 15-cm dishes. THP-1 cells were used at 4 × 10^7^ cells per sample in T75 flasks.

For coculture experiments, 5 × 10^6^ HUVEC-T1 cells were seeded in 10-cm dishes and grown for 24 h to form a confluent monolayer. SILAC-labeled A549 cells (6 × 10^6^) were detached with 10 mM EDTA disodium salt in PBS, resuspended in SILAC RPMI-1640 medium without lysine and arginine, and seeded onto the HUVEC-T1 monolayer. Cells were cocultured for 2 h during NBID labeling as described below.

### Protein Expression and Purification

Recombinant nanobodies, TurboID, and fusion constructs were expressed in *E. coli* BL21(DE3) carrying pET-28a(+)-SUMO plasmids. Starter cultures (5 ml LB + 50 μg/ml kanamycin) were grown for 8 h at 37 °C, then inoculated into 1 L fresh LB medium and cultured until OD_600_ reached 0.6 to 0.8. Expression was induced with 0.5 mM IPTG at 16 °C overnight.

Cells were harvested by centrifugation, resuspended in Tris-buffered saline (pH 7.4) with 0.2 mM PMSF, and lysed by pressure homogenization. Lysates were clarified and loaded onto Ni-NTA resin (Yeasen). Proteins were eluted with 300 mM imidazole in Tris-buffered saline, then diluted to 100 mM imidazole. SUMO tags were removed with His-tagged ULP1 protease (50 μg/ml, 3 h, 8 °C). After a further 5 × dilution, cleaved tags and protease were removed by a second Ni-NTA purification. Flow-through containing purified constructs was aliquoted and stored at −80 °C in 5% glycerol.

### Structural Prediction of Recombinant Protein Linkers

Structural models of the linker sequences were generated using the AF2Complex monomer pipeline based on AlphaFold2. Amino acid sequences of the linkers (P8: EPKIPQPA; P26: EPKIPQPQPKPQPKPEPETPKPGSGS) were used as input, and structure prediction was performed on the computing cluster of the Technology Center for Protein Sciences, Tsinghua University. Predicted structures were visualized and analyzed using ChimeraX (version 1.9). Linker lengths were estimated by measuring the distance between the N- and C-terminal atoms of the predicted models.

### NBID Labeling and Affinity Capture

#### Substrate Mix Preparation

A 50 × NBID substrate mix was prepared in water (250 mM MgCl_2_, 50 mM ATP-Na_2_, and 250 μM biotin), aliquoted, and stored at −80 °C.

#### Labeling

MU1053-P8-TurboID (3 μg/ml) was added directly to culture medium for labeling of CD38-proximal proteins. For controls, equimolar amounts of MU1053 and TurboID (nonfused) were added separately. After 15 min incubation at 37 °C, substrate mix was added, and labeling continued for 30 min. Cells were then washed three times with PBS and lysed in radioimmunoprecipitation assay buffer (Beyotime) containing protease inhibitor cocktail (Bimake). EGFR-proximal proteins were labeled using 7D12-P8-TurboID in the same manner.

For coculture experiments, NBID constructs and substrate mix were added immediately after seeding A549 cells onto HUVEC-T1 monolayers. Labeling was performed for 2 h, followed by PBS washes and lysis.

#### Affinity Capture

Lysates were sonicated, clarified by centrifugation (12,000 rpm, 15 min, 4 °C), and adjusted to 1 to 2 mg/ml total protein. Biotinylated proteins were enriched with NeutrAvidin agarose (20 μl resin per sample; Thermo Fisher Scientific) overnight at 4 °C, followed by five radioimmunoprecipitation assay washes.

### LC-MS/MS Proteomic Analysis

#### On-Beads Digestion

Captured proteins were washed 10 × with 50 mM ammonium bicarbonate. Proteins were reduced (5 mM dithiothreitol, 1 h, RT), alkylated (12.5 mM iodoacetamide, 30 min, dark), and digested on-beads with 1 μg trypsin (Promega) in 400 μl 50 mM ammonium bicarbonate at 37 °C for 16 h. After adding 1% formic acid, peptide solution was collected, vacuum-dried at 60 °C, and stored at −80 °C. Samples were reconstituted in 0.1% formic acid before LC-MS/MS.

#### Data-Independent Acquisition (DIA)

Peptides were separated by reverse-phase HPLC (75 μm i.d. × 350 mm in-house packed C18 column) and analyzed using either Orbitrap Exploris 480 (CD38 experiments) or Orbitrap Astral (EGFR experiments). Data-independent acquisition (DIA) settings were:

*Exploris 480*: MS1 400 to 1200 m/z, 120,000 resolution; DIA 200 to 2000 m/z, 400 to 1200 precursor range, 8.9 m/z isolation window, 30,000 resolution; 3 s cycle time.

*Astral*: MS1 380 to 980 m/z, 240,000 resolution; DIA 150 to 2000 m/z, 380 to 980 precursor range, 2 m/z isolation window; 0.6 s cycle (loop) time.

### Experimental Design and Statistical Rationale

#### Statistical Rationale

For EGFR PL, three technical replicates were used for each cell type to assess the validity of NBID methodology. For CD38 NBID labeling, three biological replicates were employed to ensure soundness and replicability of identified proximal candidates. For each cell type and experimental condition, protein group quantification from all PL and control samples were globally normalized during database searching by Spectronaut software to adjust for any systematic variations between different MS runs.

#### DIA Data Analysis

Data were processed in Spectronaut 18 using the directDIA workflow to search against the reviewed Uniprot database of *Homo sapiens* (Version November 2022 or December 2024, for experiments listed in “NBID Reveals the CD38-Associated Surfaceome in A549 and THP-1 Cells” or “NBID Enables Proteomic Mapping of POI-Proximal Surfaceome in Cell Culture” & “Identification of a CD38-Associated Intercellular Cadherin Adhesion Network by NBID”, respectively. Total number of entries were 20,401 and 20,422, respectively).

*DirectDIA search parameters*: *digestion* trypsin/P, *max missed cleavage* 2, *static modification* Carbamidomethyl (C), *dynamic modification* Oxidation (M) and acetylation (Protein N terminus), *mass tolerance* default. *Precursor Qvalue cutoff*, *protein Q. cutoff (run-wise)*, and *protein Q. cutoff (experiment wide)* used default values of 0.01, 0.05, and 0.01, respectively. All data from Exploris 480 were quantified using MS1 intensity and data from Astral were quantified using MS2 intensity. SILAC data were analyzed by Spectronaut with *in-silico generate missing channels* set as true and *workflow* set as label in *workflow* settings, Arg10 and Lys6 added for *Channel two* in *labeling* settings to quantify SILAC heavy labeled proteins, and *Channel one* was left blank to quantify SILAC light proteins. For SILAC channel quantifications, *major group quantity* was set as sum peptide quantity, and *major group top N* was set as false.

For CD38 experiments, six PL and control samples representing three biological replicates for each condition were analyzed collectively in Spectronaut with default normalization across all samples, with the exception of A549 monoculture for which untreated and NAD^+^ treated samples were analyzed collectively. For EGFR experiments, six samples representing three technical replicates per condition were analyzed collectively with default global normalization.

#### Post-Analysis

Output data entries were filtered by number of stripped sequences identified (≥2) and proportion of valid values across samples (≥50%, missing values, iBAQ values < 1, or SILAC channel quantities <10 were considered invalid values). Raw iBAQs or channel quantities were log2-transformed, and invalid values were imputed by sample specific (mean − 1.96 × SD).

For significance analysis, significance analysis of microarrays was performed in R (4.5.2) using *samr* package *via* its *Shiny* web interface. Number of permutations was set to 1000 (≥720 for complete enumeration in 3 *versus* three comparison) to ensure reproducibility of q-values, and the critical delta value for each dataset was determined based on the median false discovery rate from the generated delta table. Significantly enriched proximal proteins were filtered using a combination of fold-change threshold (log_2_ FC > 1) and experiment-specific q-value cutoffs, tailored to accommodate for variations in data density. Specifically, q < 0.05 was applied to EGFR experiments due to large set of significantly enriched proteins. For CD38-proximal surfaceome profiling, q < 0.1 was applied. For light channel representing HUVEC-T1 proteins in the SILAC-labeled coculture system, q < 0.2 was applied as a much more limited set of significant genes were enriched. Data were visualized in volcano plots by log_2_ FC versus abs(d) where $d\left(i\right)=\frac{\bar{x\left(i\right)}-\bar{y\left(i\right)}}{s\left(i\right)+s_{0}}$

For cellular compartment annotation of identified entries, Gene Ontology cellular compartment (GOCC) slim annotations were implemented *via* MaxQuant Perseus v2.1.4. Surface and extracellular (surface-accessible) localizations were inferred based on GOCC slim annotations being either GO:0009986 (cell surface), GO:0005576 (extracellular region), GO:0005615 (extracellular space), or GO:0005886 (plasma membrane), with the exclusion of all keratins for being potential contaminants (Supplemental Table S1). C-type mannose receptor 2 (MRC2), a known membrane protein (25) enriched in multiple of our NBID experiments but missing one of the previously mentioned GOCC annotations, was additionally included in the surface/extracellular protein list. Protein interaction/association networks were created with Cytoscape v3.10.4 with StringAPP v2.2.0 plugin (26, 27).

#### Multivariate Statistical Analysis for NAD^+^ Treatment

To assess whether NAD^+^ treatment alters altered the CD38-proximal surface proteome, multivariate statistical analyses were performed on combined proteomic datasets from untreated (WT) and NAD^+^-treated A549 cells, each consisting of three PL samples and three control samples. Protein groups were filtered in MaxQuant Perseus as described previously (≥2 unique stripped sequences and ≥50% valid values in either WT or NAD^+-^treated samples). LFQ intensities were log2-transformed, missing values were imputed, and surface-accessible proteins were retained.

To focus on proteins contributing most strongly to sample-to-sample variation, the top 100 variable surface-associated proteins were selected based on mean absolute deviation. The resulting protein matrix was exported to R for downstream multivariate analyses.

Pearson correlation distance (1 − r) was calculated between samples and used for hierarchical clustering, principal coordinate analysis (PCoA), and permutational multivariate analysis of variance (PERMANOVA). Hierarchical clustering heatmaps were generated using the *pheatmap* package with complete-linkage clustering applied to both proteins and samples. PCoA was performed using the same Pearson correlation distance matrix. Global PERMANOVA was performed using the *adonis2* function in the *vegan* package with 9999 permutations. Pairwise PERMANOVA comparisons were subsequently conducted between selected groups. Homogeneity of multivariate dispersion was assessed using the *betadisper* function prior to PERMANOVA analysis.

### Transient Transfection and Total Internal Reflection Fluorescence (TIRF) Microscopy

A549 cells were seeded into 35-mm glass-bottom dishes 24 h before transfection. Cells were cotransfected with CD38-FP fusion constructs and FP-fused proximal proteins identified by NBID (CDH2, CRIM1, FZD2, NECTIN2, or PRNP) using Lipofectamine 3000 (Invitrogen) according to manufacturer’s instructions. After 48 h, cells were imaged on a Nikon TIRF microscope with a 100 × SR HP Apo TIRF 1.49 NA oil objective.

### TEM Assay

HUVEC-T1 cells (4 × 10^5^ cells per insert) were seeded onto Transwell inserts (6.5 mm diameter, 8.0 μm pore size; Corning 3422) and cultured for 48 h to form confluent endothelial monolayers.

A549 cells were labeled with CFDA-SE (4 μM; ApexBio) according to the manufacturer's instructions. Endothelial monolayers were incubated with recombinant human CD38 protein (10 μg/ml) or bovine serum albumin (BSA) control for 1 h at 37°C and subsequently washed with PBS.

Labeled A549 cells (4 × 10^5^ cells per insert) suspended in serum-free RPMI-1640 medium were added to the upper chamber, while the lower chamber contained RPMI-1640 supplemented with 10% FBS as attractant. Following 9 h incubation, cells that had migrated to the lower compartment were collected by trypsinization and resuspended in equal volumes of PBS.

Migrated CFDA-SE-positive cells were quantified using a BD LSRFortessa SORP flow cytometer, with CFDA-SE fluorescence detected in the FITC channel. Samples were acquired using identical instrument settings, a fixed medium flow rate, and a fixed acquisition time of 60 s for all samples. The number of CFDA-SE-positive events acquired during this interval was used as a relative measure of transmigrated cell abundance. Statistical significance was assessed using a two-sided Student's *t* test.

## Results

### Expression and Validation of NTCs for PL

**Fig. 1:**
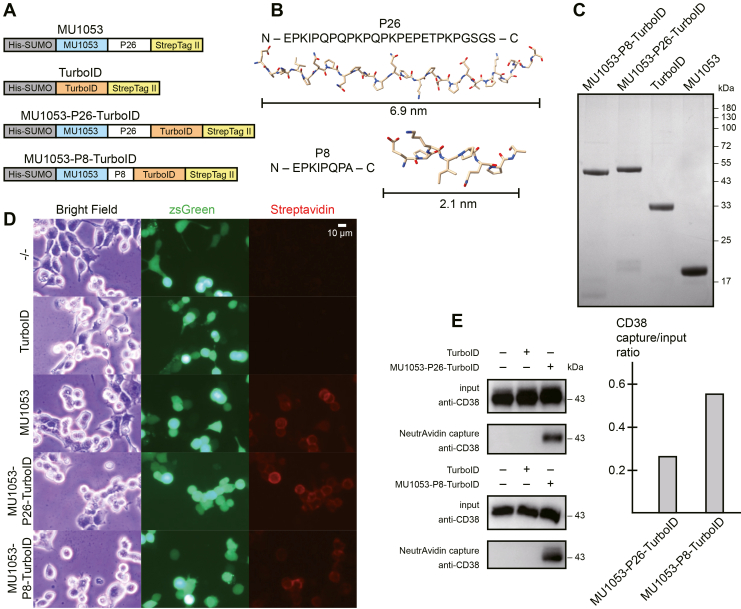
**Design, expression, and validation of nanobody–TurboID fusion proteins for NBID proximity labeling.***A*, recombinant protein constructs for CD38 NBID labeling. All constructs include an N-terminal His-SUMO tag (*gray*), which is then removed during purification, and a C-terminal StrepTag II (*yellow*) for detection. The CD38-targeting nanobody MU1053 (*blue*) is fused to TurboID (*orange*) *via* one of two rigid linkers: P26 or P8. TurboID and MU1053 were also expressed individually for controls. *B*, structural models of the two linker peptides by AlphaFold. P26 consists of a 26-residue proline-rich hinge-like sequence (∼6.9 nm), and P8 is a truncated version (∼2.1 nm), both derived from the camelid HcAb hinge region. *C*, SDS-PAGE analysis of purified recombinant proteins expressed in *E. coli*. *D*, fluorescence microscopy of CD38-transfected HEK293T cells incubated with indicated recombinant proteins. Cells express zsGreen1 (*middle panels*) as a transfection marker. Streptavidin staining (*right panels*) reveals specific binding of MU1053-containing constructs to CD38-positive cells. The background control was not incubated with any of the recombinant proteins and only incubated with fluorescent streptavidin. *E*, Western blot analysis of CD38 labeling efficiency. HEK293T cells transfected with CD38 were labeled with MU1053-P26-TurboID or MU1053-P8-TurboID in the presence of biotin, followed by NeutrAvidin pull-down and immunoblotting against CD38. Quantification of band intensity (pulldown/input ratio) shows greater labeling efficiency for the shorter P8 linker construct. CD38, cluster of differentiation 38; NBID, nanobody-targeted TurboID; SUMO, small ubiquitin-like modifier.

To enable nanobody-guided PL, we designed and expressed NTCs consisting of the CD38-targeting nanobody MU1053 fused to the promiscuous biotin ligase TurboID. Two different chimeras were constructed with rigid, proline-rich linker sequences of different lengths—P26 (26 residues, ∼6.9 nm) and P8 (8 residues, ∼2.1 nm), both derived from truncated camelid HcAb hinge—positioned between the nanobody and TurboID (Fig. 1, *A* and *B*). All constructs, including individual nanobody and TurboID constructs, included an N-terminal His-SUMO tag for enhanced solubility (removed during purification) and a C-terminal StrepTag II for detection.

The protein constructs were expressed in *E. coli* and purified by Ni-NTA affinity chromatography. SDS-PAGE analysis showed high-purity bands corresponding to the expected molecular weights for MU1053-P26-TurboID, MU1053-P8-TurboID, TurboID, and MU1053 nanobody (Fig. 1*C*). The yields of each construct exceeded 10 mg per liter of bacterial culture, supporting the scalability of this approach.

To confirm specific POI binding, we incubated CD38-transfected HEK293T cells with the recombinant proteins and stained with fluorescent streptavidin. MU1053, MU1053-P26-TurboID, and MU1053-P8-TurboID showed strong surface staining in CD38-positive cells, while TurboID alone exhibited minimal signal compared with background control (Fig. 1*D*). All constructs showed minimal binding to mock-transfected HEK293T cells (not shown).

We next compared the labeling efficiency of the two CD38-targeting NTCs. CD38-transfected HEK293T cells were incubated with either MU1053-P26-TurboID or MU1053-P8-TurboID in the presence of biotin. Biotinylated proteins were captured with NeutrAvidin resin and analyzed by Western blot. Both constructs successfully labeled CD38, but the shorter P8 linker yielded a higher CD38 enrichment relative to input (capture/input ratio: ∼0.55 *versus* ∼0.27), indicating improved PL efficiency (Fig. 1*E*). TurboID alone does not biotinylate CD38 under the same conditions.

Based on these results, the P8 linker was selected as the optimal configuration for NTCs used in subsequent PL experiments. Using the same purification workflow, we additionally purified an EGFR-targeting NTC using nanobody 7D12, which is widely used in cancer diagnostic and therapeutic studies (28), to validate the NBID-based proximal proteome approach by comparison with the established EGFR-associated protein network.

### NBID Enables Proteomic Mapping of POI-Proximal Surfaceome in Cell Culture

**Fig. 2:**
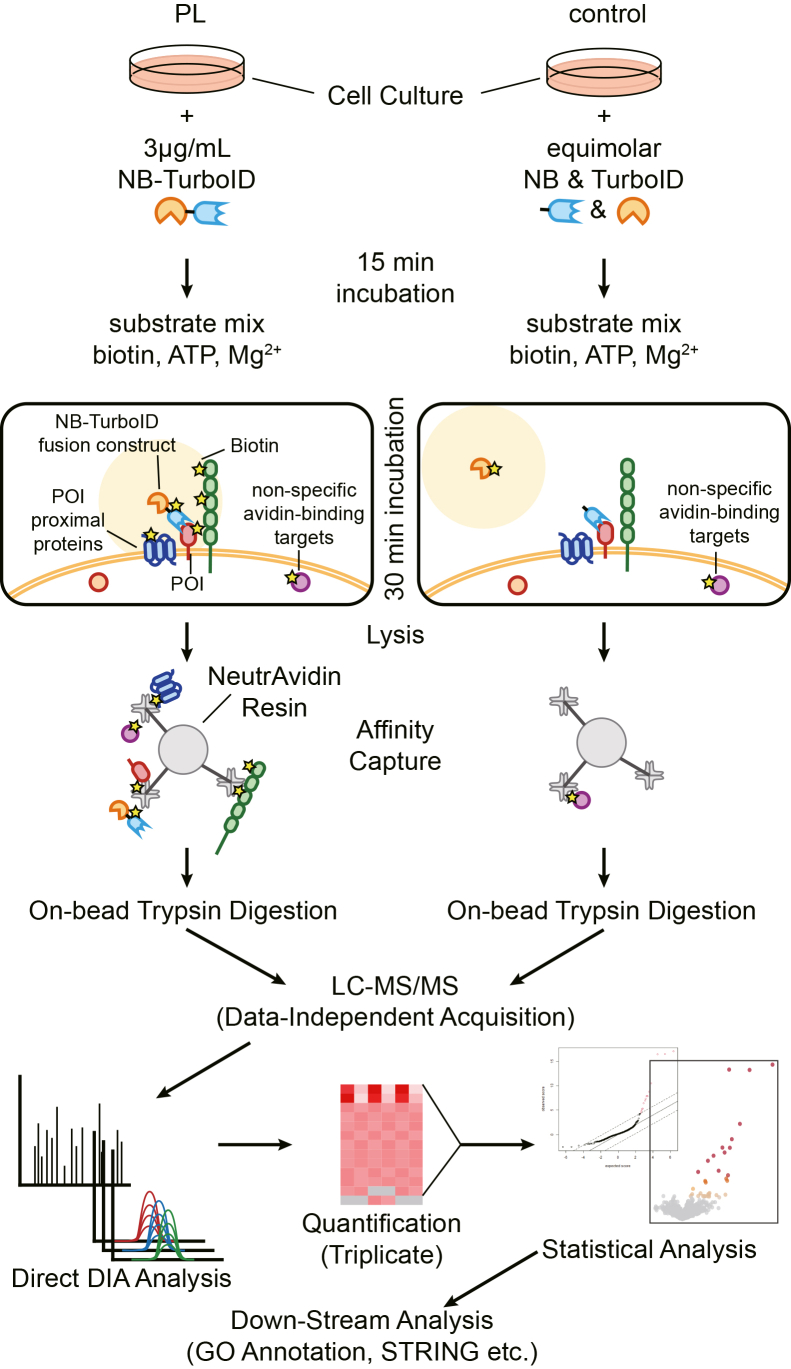
**Schematic workflow of NBID-based proximity labeling for mapping the CD38 proximal surfaceome.** Cell cultures were incubated with POI-specific NTCs (proximal labeling, PL) or an equimolar mixture of nanobody and TurboID (control). After 15 min of binding, proximity labeling was initiated by the addition of biotin, ATP, and Mg^2+^. Following cell lysis, biotinylated proteins were captured using NeutrAvidin resin and digested on-bead with trypsin. Peptides were analyzed by DIA-based LC-MS/MS and quantified across biological triplicates. Enriched proteins were identified by SAM and interpreted with downstream annotation and interaction network analysis. CD38, cluster of differentiation 38; DIA, data-independent acquisition; NBID, nanobody-targeted TurboID; SAM, significance analysis of microarrays.

To validate the reliability of NBID for identifying surface protein neighborhood in live cell culture, we included EGFR—a surface protein with a well-characterized interaction network—as a model POI, as current knowledge to the CD38 associated proteins remains far less comprehensive. We applied the 7D12 NTC to adherent A549 and HeLa cell cultures to profile their EGFR-associated surface protein networks. The experimental setup for the NBID assay is illustrated in Figure 2 and described in detail in the Experimental Procedures. Briefly, NTC was added to the cell cultures, and a substrate mixture was supplied to induce proximity-based biotinylation of proteins surrounding the POI, while an equimolar mixture of separate nanobody and TurboID replaced the NTC in control samples to account for nonspecific background.

**Fig. 3:**
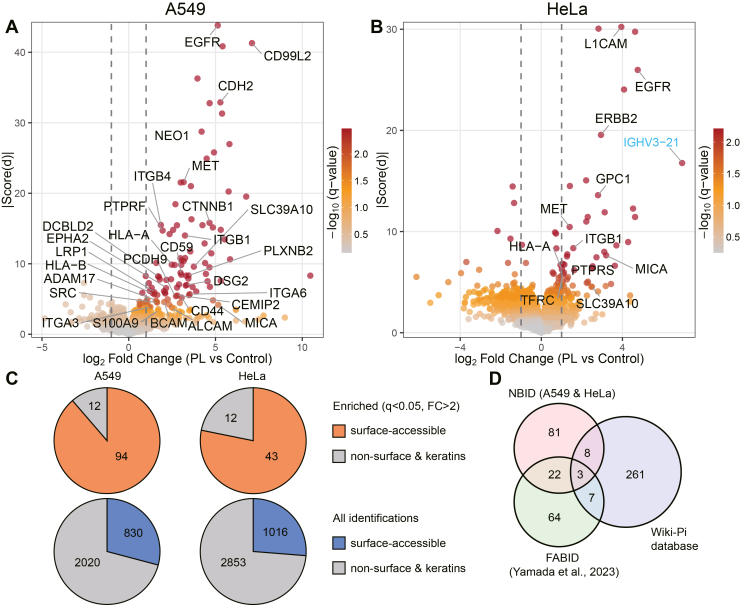
**NBID identified EGFR proximal proteome.***A* and *B*, volcano plots showing NBID enriched EGFR proximal proteome in A549 (*A*) and HeLa (*B*) cells, with known interactors highlighted. Horizontal axis: log_2_ fold change (PL *versus* control); vertical axis: absolute *d*-score from the SAM test; significance threshold: fold change >2, *q* < 0.05; color scale: −log10 *q*-value. IGHV3-21 identification highlighted in *cyan* (*B*) originates from the nanobody sequence. *C*, pie chart showing the fraction of surface-accessible, non-keratin proteins from significantly enriched proteins or all identified proteins from respective cell type. *D*, Venn diagram showing overlap of NBID identified EGFR-proximal proteome and literature-based interactors (FabID & Wiki-Pi database).EGFR, epidermal growth factor receptor; NBID, nanobody-targeted TurboID; PL, proximity labeling; SAM, significance analysis of microarrays.

For NBID samples from A549 and HeLa cells, DIA-based LC-MS/MS identified 2850 and 3869 proteins, respectively, each with at least two unique peptides and ≥50% completeness (ratio of valid quantification across samples). EGFR was robustly enriched in all PL samples (Fig. 3, *A* and *B*, Supplemental Tables S2 and S3). Most proteins showed no significant enrichment in PL samples, representing the baseline signal from non-proximal labeled control samples. Among the identified proteins, 106 and 55 proteins in A549 and HeLa cells, respectively, showed significant enrichment (fold change >2 and *q* < 0.05), with the majority annotated as surface or extracellular proteins (Fig. 3*C*). In total, NBID identified 114 distinct EGFR-proximal, surface-associated proteins, including 33 previously reported interactors (highlighted in Fig. 3, *A* and *B*) documented in the Wiki-Pi database (https://hagrid.dbmi.pitt.edu/wiki-pi/index.php/, (29)) and in a recent EGFR-proximal proteomics study that used AirID-conjugated antibody fragments (FABID, (30)) (Fig. 3*D*). Taken together, these results indicate that NBID is a reliable tool for proteomic characterization of POI-associated surfaceome networks in living cells.

### NBID Reveals the CD38-Associated Surfaceome in A549 and THP-1 Cells

To characterize the CD38-associated surface proteome, we applied the MU1053 NTC to A549 (non–small cell lung cancer) and THP-1 (human monocytic leukemia) cells, both of which express high levels of CD38 on the cell surface. For A549 cells, we additionally included a condition with 1 μM NAD^+^ treatment during NBID labeling to evaluate whether the acute exposure to CD38 substrate altered the CD38-proximal surfaceome.

**Fig. 4:**
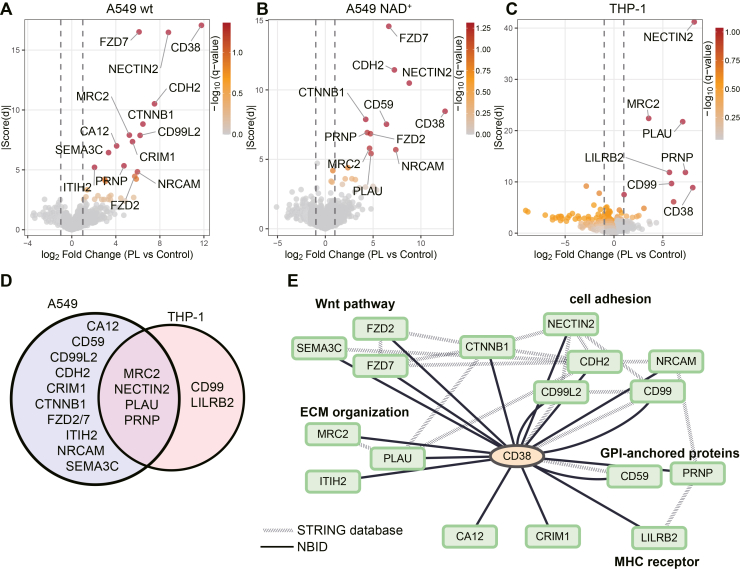
**NBID reveals CD38-associated surfaceome in A549 and THP-1 cells.***A*, *B*, and *C*, volcano plots showing proteins enriched by NBID in WT (untreated) A549 cells (A), A549 cells treated with 1 μM NAD^+^ (*B*), and THP-1 cells (*C*). Horizontal axis: log_2_ fold change (PL *versus* control); vertical axis: absolute *d*-score from the SAM test; significance threshold: fold change >2, *q* < 0.1; color scale: −log10 *q*-value. *D*, Venn diagram showing the overlap of CD38-proximal proteome between A549 and THP-1 cells. *E*, NBID identified CD38-proximal proteome in A549 and THP-1 cells integrated with the STRING network. Proteins are clustered by literature- and Uniprot-based functional associations. CD38, cluster of differentiation 38; NBID, nanobody-targeted TurboID; PL, proximity labeling; SAM, significance analysis of microarrays; THP-1, Tohoku Hospital Pediatrics-1.

Across all cell types and conditions, CD38 was robustly enriched in all PL samples, indicating efficient biotinylation of the POI by the MU1053 NTC (Fig. 4, *A*–*C*). In untreated (wt) A549 cells, NBID identified 1758 proteins with at least two unique peptides and ≥50% completeness across three biological replicates (Fig. 4*A*, Supplemental Table S4), with significant enrichment (fold change >2, *q* < 0.1) of 13 proteins apart from CD38. In NAD^+^ treated A549 cells, 10 proteins apart from CD38 were significantly enriched among 1710 valid identifications (Fig. 4*B*, Supplemental Table S5). All significantly enriched proteins bear surface and/or extracellular GOCC annotations, indicating minimal off-target enrichment of intracellular proteins under the applied statistical thresholds. In THP-1 cells, six surface-accessible proteins were significantly enriched (fold change >2, *q* < 0.1), apart from CD38 and a keratin contaminant, among 1919 valid identifications (Fig. 4*C*, Supplemental Table S6).

**Supplemental Figure S1:**
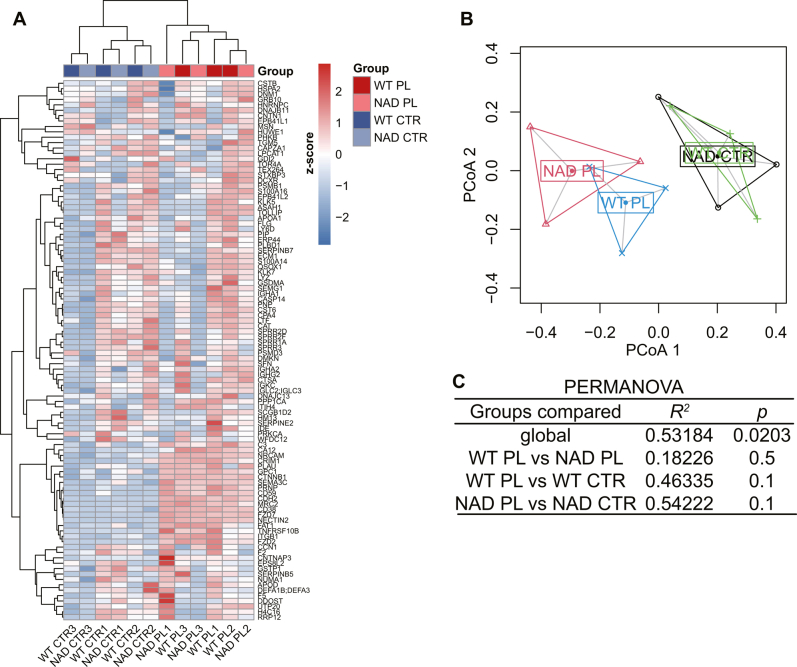
**Supplemental figure S1. Global comparison of CD38 proximity labeling datasets from untreated (WT) and NAD+ treated A549 cells.***A*, Hierarchical clustering heatmap of the top 100 most variable surface-associated proteins identified across all 12 A549 samples. Samples primarily clustered according to proximity labeling status (PL vs CTR), whereas no clear separation was observed between untreated (WT) and NAD+-treated (NAD) samples. *B*, Principal coordinate analysis (PCoA) of the same protein set. PL and CTR samples formed distinct clusters, while WT and NAD samples showed substantial overlap within the PL and CTR groups. *C*, Global comparison among all groups showed significant separation by PERMANOVA, while paired comparison between WT PL and NAD PL groups showed no statistically significant separation. Sample/group names: WT PL: untreated proximity-labeled; WT CTR: untreated control; NAD PL: NAD+ treated proximity-labeled; NAD CTR: NAD+ treated control.

The enriched CD38-proximal proteomes from wt or NAD^+^ treated A549 cells largely overlap. To assess global variance between the two treatments, hierarchical clustering and PERMANOVA were performed for top-variable surface-accessible protein identifications across all A549 samples (Supplemental Fig. S1). Overall, hierarchical clustering and PCoA showed no apparent separation between WT and NAD^+^ treated PL samples whereas PL and control samples were clearly separated under different clusters (Supplemental Fig. S1, *A* and *B*). PERMANOVA test showed significant global difference among all groups (*R*^*2*^ = 0.53, *p* = 0.02), while no significant distinction between wt and NAD^+^ treated PL groups (*R*^*2*^ = 0.18, *p* = 0.5) (Supplemental Fig. S1*C*). Together, these analyses indicate that acute NAD+ treatment does not significantly alter the CD38-proximal protein network.

Among all enriched CD38-proximal proteins, PRNP, NECTIN2, MRC2, and urokinase-type plasminogen activator (PLAU) are common to both A549 and THP-1 cells. Notably, MRC2 (endocytic receptor 180), PLAU, and the A549-specific inter-alpha-trypsin inhibitor heavy chain H2 are known ECM modulators involved in EMT and invasion of cancer cells (25, 31, 32). In our experiments, a variety of cell adhesion proteins were also enriched in the CD38-proximal neighborhood, including CD99, CD99 antigen-like protein 2, CDH2, NECTIN2, and neuronal cell adhesion molecule. Such proteins mediate a variety of biological processes such as cell-cell adhesion, T-cell activation, leukocyte transmigration, cancer metastasis, and synapse formation through intercellular interactions and signaling (33, 34, 35, 36, 37). We also identify the Wnt signaling receptors FZD2 (Frizzled-2) and FZD7 (Frizzled-7) (38), and semaphorin-3C (SEMA3C), a reported positive regulator of Wnt/β-catenin signaling (39), specifically in A549 cell culture, suggesting that CD38 could be spatially linked to the Wnt signalosome. We also notice the enrichment of catenin beta-1 (CTNNB1), the intracellular cytoskeletal scaffold of the cadherin–catenin complex and the key downstream signaling transductor of the Wnt pathway, likely coenriched through its stable association with the cadherin–catenin complex that persists during detergent-based cell lysis. In THP-1 cells, the enrichment of the MHC receptor LILRB2 suggests that CD38 may participate in membrane microdomains involved in immune signaling.

In summary, NBID combined with DIA LC-MS/MS successfully identified cell-type-specific CD38-proximal proteins in our target cell models with high confidence, while NAD^+^ treatment had minimal immediate impact to the CD38-proximal neighborhood in A549 cells. To visualize functional relationships among identified proteins, we integrated NBID-derived CD38-proximal proteome with STRING-reported protein–protein associations (Fig. 4*E*). The resulting network revealed clusters associated with cell adhesion, ECM organization, and Wnt signaling. In our previous study, CDH2 and CTNNB1 were up-regulated following CD38 overexpression through NAD^+^-decline-induced EMT (14). The current findings extend these observations by suggesting that CD38 is not only functionally linked to these proteins but may also reside in close physical proximity to the cadherin–catenin complex. In A549 cells, the enrichment of adhesion molecules, ECM regulators, and Wnt-associated proteins suggests that CD38 may contribute to the organization of membrane microdomains involved in cell adhesion, ECM remodeling, and metastatic behavior.

### Coclustering of CD38 and NBID-Identified Interactors on the A549 Cell Surface

**Fig. 5:**
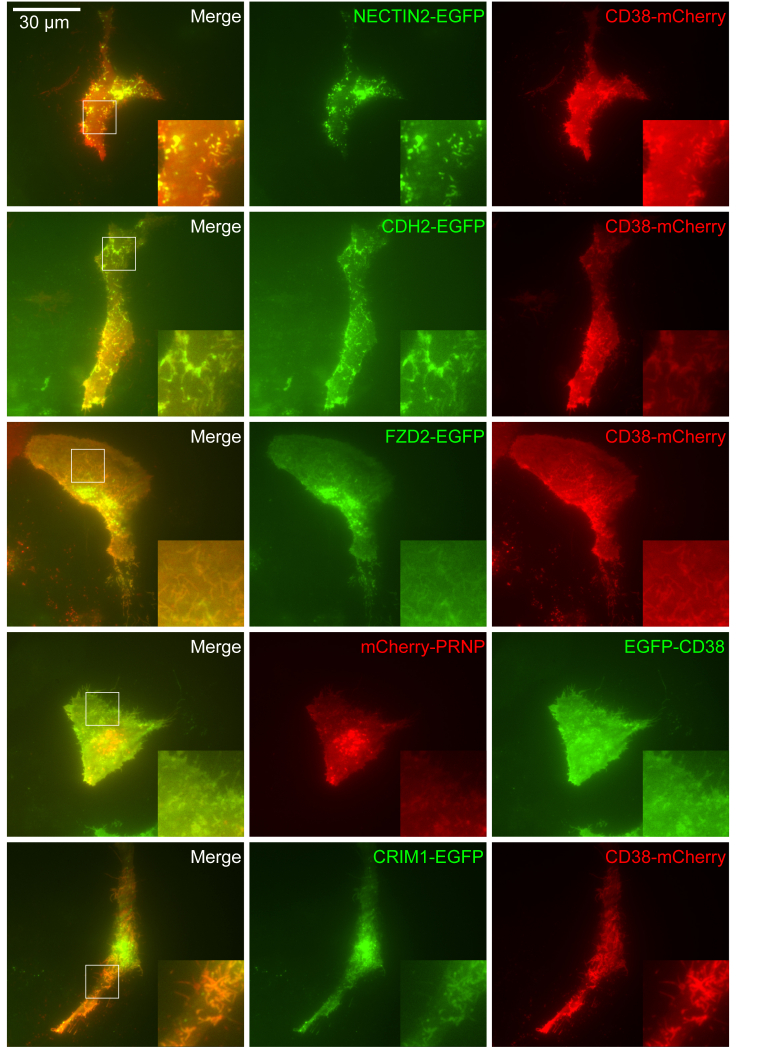
**Coclustering of CD38 with NBID-identified interactors on the A549 cell surface.** A549 cells were co-transfected with fluorescent protein (FP) fusions of CD38 and five NBID-identified proximal proteins (NECTIN2, CDH2, FZD2, PRNP, and CRIM1) and imaged by total internal reflection fluorescence microscopy (TIRFM). Representative images show merged channels (*left*), individual interactor channels (*middle*), and CD38 (*right*). Insets highlight regions of interest indicated by *white boxes*. NECTIN2, CDH2, and FZD2 exhibited clear coclustering with CD38 at the plasma membrane, particularly on surface protrusions. Significant signal from PRNP and CRIM1 FP fusion was detected in near-surface vesicles but they still showed surface coclustering with CD38 on similar protrusions. The scale bar represents 30 μm. CD38, cluster of differentiation 38; CDH2, N-cadherin; FZD2, frizzled class receptor 2; CRIM1, cysteine-rich transmembrane BMP regulator 1; FP, fluorescent protein; NBID, nanobody-targeted TurboID; NECTIN2, nectin-2; PRNP, prion protein.

To validate the CD38-proximal proteins identified by NBID, we transfected A549 cells with FP fusion constructs of CD38 and five high-confidence NBID identifications with strong MS coverage—NECTIN2, CDH2, FZD2, PRNP, and CRIM1—and visualized their surface distribution using total internal reflection fluorescence microscopy (TIRFM), which selectively detects fluorescence within a few hundred nanometers of the glass–aqueous interface (40). TIRFM revealed that four candidates—NECTIN2, CDH2, FZD2, and PRNP—showed pronounced coclustering with CD38 on the cell surface. CRIM1 localized primarily to near-surface cytoplasmic vesicles, likely due to impaired trafficking caused by the FP fusion, but still exhibited partial coclustering with CD38 at the plasma membrane (Fig. 5).

Notably, CD38 and its coclustering partners were enriched on filamentous, pseudopod-like protrusions resembling filopodia or invadopodia—structures associated with cellular adhesion, ECM interaction, and motility. This observation is consistent with the NBID proximity proteomics results, which identified multiple surface proteins involved in related membrane structures and functions. Together, these data, along with reports linking CD38 expression to poor prognosis in certain tumor types (4), lead us to hypothesize that CD38 may contribute to tumor cell migration. We examine this possibility in the following section using A549 cells.

### CD38-Mediated Interactions Contribute to A549 TEM

Our PL analysis identified multiple CD38-proximal proteins involved in cell adhesion and membrane organization. In addition, CD38 has previously been reported to interact with the endothelial adhesion molecule PECAM-1 (CD31) to promote leukocyte adhesion and TEM (9). We therefore investigated whether CD38-mediated interactions may contribute to TEM in solid tumor cells using the A549 cell line.

**Fig. 6:**
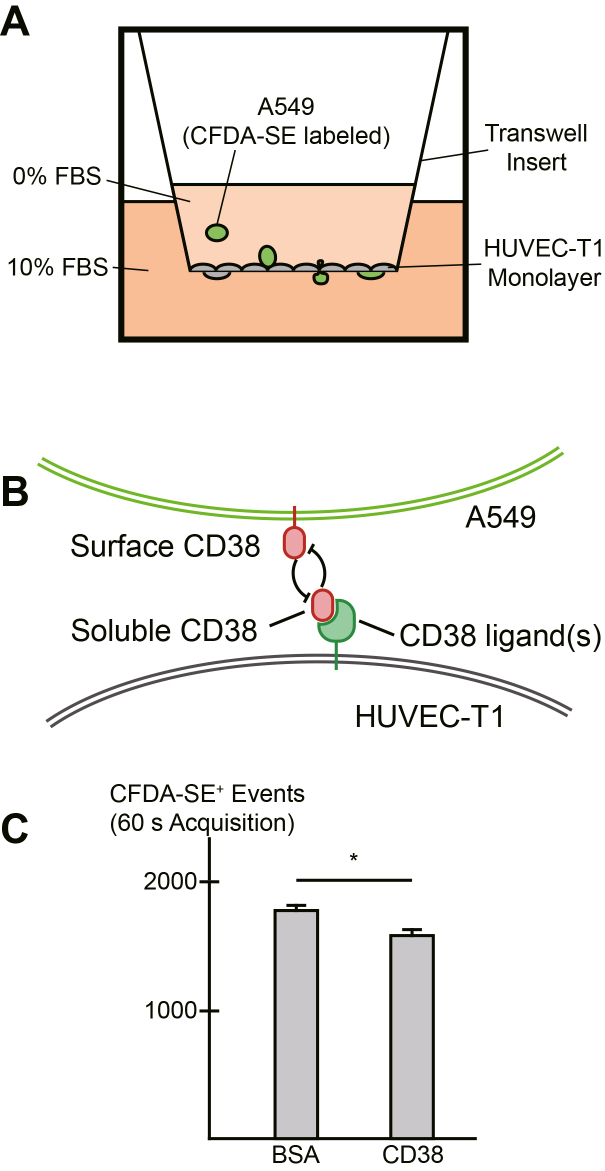
**CD38 modulates A549 cell transendothelial migration.***A*, schematic illustration of the transendothelial migration assay. HUVEC-T1 monolayers established on transwell inserts were pretreated with soluble CD38 protein or BSA (control) before seeding with CFDA-SE-labeled A549 cells. Transendothelial migration was induced across a fetal bovine serum (FBS) gradient (0% to 10% FBS). Migrated cells on the lower surface of the inserts were trypsinized and quantified by flow cytometry. *B*, hypothetical model illustrating a potential mechanism by which soluble CD38 interferes with CD38-mediated interactions between A549 cells and endothelial cells. *C*, relative abundance of transmigrated A549 cells (CFDA-SE-positive events per 60 s acquisition) showing a significant reduction in migration following soluble CD38 treatment of the endothelial monolayer compared to the BSA control. Data are presented as mean ± SEM (n = 3 biological replicates), two-sided Student's *t* test (*p* = 0.034). BSA, bovine serum albumin; CD38, cluster of differentiation 38; HUVEC-T1, SV40T transfected human umbilical vein endothelial cell.

A HUVEC-T1 endothelial monolayer was established in Transwell inserts and used as an *in vitro* TEM model (Fig. 6, *A* and *B*). To interfere with potential CD38-mediated interactions at the endothelial surface, monolayers were preincubated with recombinant soluble CD38 protein prior to the addition of fluorescently labeled A549 cells. After serum-driven migration, the abundance of transmigrated cells was relatively quantified by flow cytometry under identical acquisition settings.

Our results show that preincubation with soluble CD38 resulted in a significant reduction in A549 TEM compared with the BSA-treated control (11.0% decrease, *p* = 0.034; Fig. 6*C*, Supplemental File S7). These findings suggest that endothelial CD38-binding molecules contribute to A549 TEM and support a potential role for CD38-mediated cell–cell interactions at the tumor–endothelium interface.

### Identification of a CD38-Associated Intercellular Cadherin Adhesion Network by NBID

Having demonstrated that CD38 contributes to A549 TEM, we next sought to identify intercellular protein complexes physically associated with CD38 during tumor–endothelial interaction. To this end, we combined NBID labeling with SILAC in a tumor–endothelial coculture system. SILAC heavy-labeled A549 cells were seeded onto a SILAC light HUVEC-T1 monolayer, after which the MU1053 NTC or the NB + TurboID control mixture, together with substrate, was immediately added to initiate PL. The labeling duration (2 h) was selected to allow A549 cells to fully settle and establish contact with the endothelial monolayer.

**Fig. 7:**
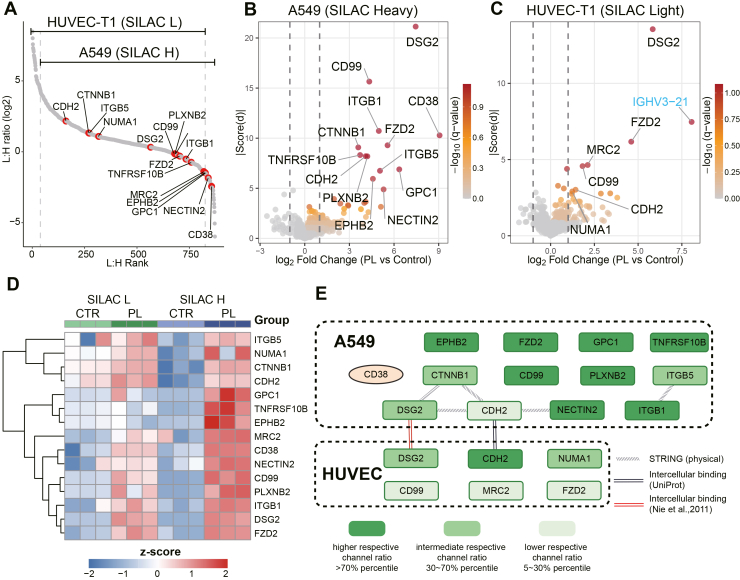
**Intercellular CD38 interaction network between A549 and HUVEC-T1 cells identified by NBID.***A*, distribution of log_2_ SILAC Light:Heavy (L:H) ratios across ranked proteins identified in A549 (heavy channel) and HUVEC-T1 (light channel) cocultures. *Top brackets* indicate the cell-type assignment ranges for HUVEC-T1 (SILAC L) and A549 (SILAC H), with cutoff based on 95% quantile. *Red circles* highlight significantly enriched proteins. *B* and *C*, Volcano plots showing CD38-proximal proteome enriched in the heavy (A549-derived, *B*) and light (HUVEC-T1–derived, *C*) SILAC channels. Horizontal axis: log_2_ fold change (PL *versus* control); vertical axis: absolute *d*-score from the SAM test; significance threshold: fold change >2, *q* < 0.1 (A549) or q < 0.2 (HUVEC-T1); color scale: −log10 *q*-value. The IGHV3-21 identification highlighted in *cyan* (*C*) originates from the nanobody sequence. *D*, hierarchical clustering heatmap showing the relative abundance (*z*-score) of significant proximal proteins across control (CTR) and proximity-labeling (PL) conditions within both SILAC channels. *E*, string- and literature-based interactions between identified CD38-proximal proteome from both cell types. CD38, cluster of differentiation 38; HUVEC-T1, SV40T transfected human umbilical vein endothelial cell; NBID, nanobody-targeted TurboID; PL, proximity labeling; SILAC, stable isotope labeling by amino acids in cell culture.

DIA-based MS analysis identified 872 proteins with at least two unique peptides and ≥50% completeness across biological triplicates. Proteins were ranked according to their average log2-transformed SILAC light:heavy (L:H) ratios. The resulting distribution followed a near-Gaussian pattern, and we assigned the upper and lower 95% quantiles to HUVEC-T1- and A549-derived proteins, respectively (Fig. 7*A*; Supplemental Table S8).

To identify proteins specifically enriched by NBID labeling, proteins assigned to each cell type according to their L:H ratios were analyzed using SAM in the corresponding SILAC channel. Proteins with fold change >2 and q-value <0.1 in the A549 channel or q-value <0.2 in the HUVEC-T1 channel were considered significantly enriched (Fig. 7, *B* and *C*; Supplemental Tables S9 and S10). The enriched CD38-proximal proteins were expected to originate from either A549 cell surface or from the A549–HUVEC-T1 interface, as the HUVEC-T1 cell line does not express detectable level of CD38 (Supplemental Table S11). Heatmap of significant proteins showed consistent enrichment in proximity-labeled samples relative to controls within their respective SILAC channels (Fig. 7*D*).

In the A549-derived (heavy) channel, 13 proteins were significantly enriched, including CD38, DSG2, CDH2, CTNNB1, ITGB1, ITGB5, CD99, FZD2, PLXNB2, GPC1, EPHB2, MRC2, and TNFRSF10B. Most of these proteins overlapped with CD38-proximal candidates previously identified in A549 monocultures, whereas DSG2, PLXNB2, and EPHB2 were specifically enriched in the coculture setting. In the HUVEC-T1-derived (light) channel, six proteins met the significance criteria—DSG2, FZD2, MRC2, CD99, CDH2, and NUMA1. Compared with the A549 cell channel, endothelial-derived proteins generally exhibited lower enrichment levels, consistent with the expectation that only a fraction of CD38 molecules on A549 cells directly participate in tumor–endothelial contacts.

Notably, DSG2 and CDH2 were significantly enriched in both A549 and HUVEC-T1 cells. As DSG2 and CDH2 are members of the cadherin superfamily and CTNNB1 was additionally enriched in the A549 channel, these findings suggest that CD38 may be incorporated into cadherin-associated intercellular adhesion complexes during tumor–endothelial contact. Consistent with this interpretation, STRING- and literature-based interaction analysis revealed an interconnected physical interaction network associating DSG2, CDH2, and CTNNB1 (Fig. 7*E*). Previous studies have shown that DSG2 can participate in both homophilic adhesion and heterophilic interactions with desmocollins (DSCs) (41, 42). In our dataset, DSC1 and DSC3 were detected but were not significantly enriched in either SILAC channel (Supplemental Tables S9 and S10). While this observation does not exclude heterophilic interactions with other partners, it suggests that the DSG2 enrichment observed at the tumor–endothelial interface may not primarily arise from canonical DSG–DSC interactions and could instead be consistent with homophilic DSG2-mediated adhesion. PLXNB2 was also specifically enriched in cocultures and may be recruited through interaction with its known ligand SEMA3C (43), which was identified as a CD38-proximal protein in the monoculture experiment (Fig. 4).

**Supplemental Figure S2:**
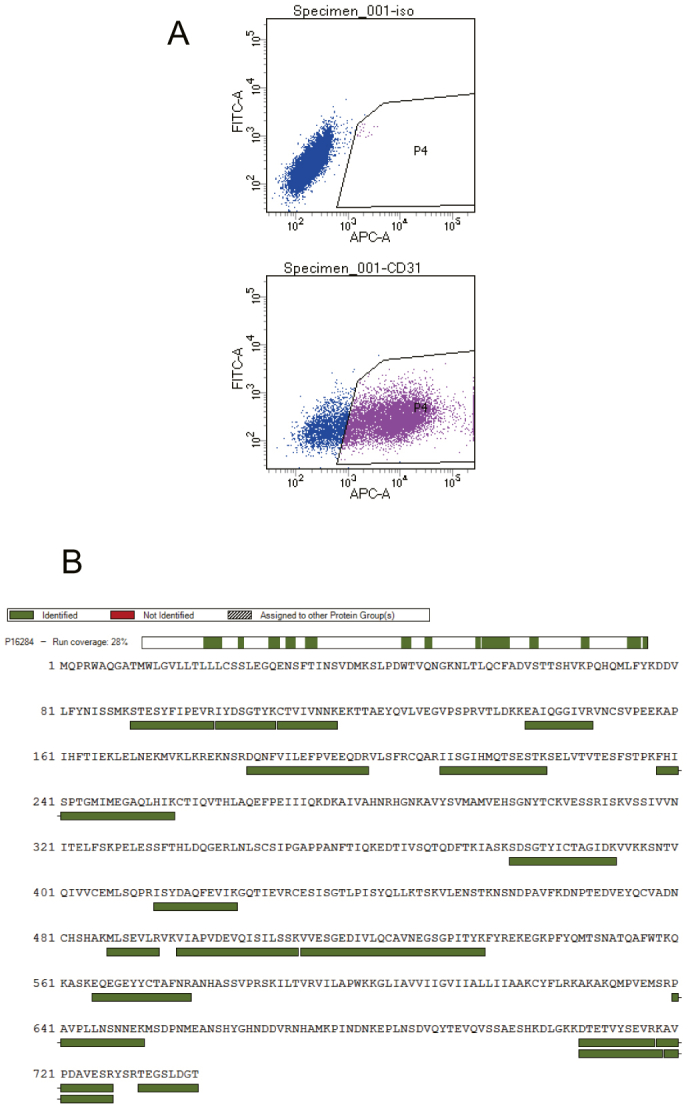
**Supplemental figure S2. PECAM-1 expression in HUVEC-T1 cells.** Flow cytometry (*A*) and DIA-based LC-MS/MS (*B*) confirmed PECAM-1 expression in HUVEC-T1 cells. (*A*) Upper: isotype; lower: anti–PECAM-1; (*B*) DIA coverage of PECAM-1 from HUVEC-T1 cell lysate.

Interestingly, PECAM-1, the previously reported CD38 binding partner (9), was not detected in any NBID-labeled sample despite its confirmed expression in HUVEC-T1 cells (Supplemental Fig. S2; Supplemental Table S11). This observation may indicate that CD38–PECAM-1 interactions are absent, transient, or not efficiently labeled under the present experimental conditions. Alternatively, binding of the MU1053 nanobody may sterically interfere with potential CD38–PECAM-1 interactions. Further studies will be required to distinguish among these possibilities.

Taken together, NBID-based coculture proteomics identified a reproducible set of proteins associated with CD38 at the tumor–endothelial interface. The bilateral enrichment of DSG2 and CDH2, together with the associated adhesion network identified in both interacting cell types, supports a model in which CD38 is incorporated into cadherin-associated intercellular adhesion complexes during tumor–endothelial contact. Given the established roles of DSG2, CDH2, and CTNNB1 in cell adhesion, migration, and EMT, these findings provide a potential molecular framework linking CD38 to the adhesive processes underlying TEM of tumor cells.

## Discussion

In this study, we established and applied a nanobody-targeted PL strategy to investigate the membrane environment of CD38 in native cell cultures. NBID identified cell type-specific CD38-proximal proteomes across multiple experimental systems, including A549 and THP-1 monocultures as well as SILAC-labeled A549–HUVEC-T1 cocultures. Several highly enriched candidates were further validated by TIRFM, which revealed their coclustering with CD38 in filamentous membrane protrusions. Together, these observations support the notion that CD38 resides within cell type-specific membrane protein assemblies that contribute to its biological functions (11, 12). We additionally demonstrated that CD38 participates in A549 TEM through interactions with endothelial cells, although the specific molecular ligands involved remain unresolved.

In A549 cells, the CD38-proximal proteome was enriched in proteins involved in cell adhesion (CDH2, CD99 antigen-like protein 2, neuronal cell adhesion molecule, and NECTIN2), Wnt signaling (FZD2, FZD7, and SEMA3C), and ECM organization (MRC2, PLAU, and inter-alpha-trypsin inhibitor heavy chain H2) (Fig. 4). In addition, the enrichment of the GPI-anchored proteins PRNP and CD59 is consistent with previous studies linking CD38 to lipid raft-associated membrane microdomains (12). The biological implications of these observations are particularly intriguing in light of the long-standing question of how CD38 exerts receptor-like functions. Unlike classical signaling receptors, CD38 possesses only a short cytoplasmic tail and lacks recognizable signaling motifs. Previous studies have therefore proposed that CD38 functions through lateral association with neighboring membrane proteins and through its incorporation into membrane microdomains, exemplified by the CD38–CD19–CD81 signaling complex in B cells (12). Our findings are consistent with this model and support the existence of a CD38-associated membrane adhesion network. Interestingly, NAD^+^ treatment did not induce major changes in the CD38-proximal surface proteome (Supplemental Fig. S1). Although this observation does not exclude potential role for CD38 as a cell-surface NAD^+^ sensor, the predominance of adhesion-associated proteins directed our attention toward potential functions of CD38 in nonsubstrate interactions. This interpretation was further supported by TIRFM observations showing preferential localization of CD38 and several validated candidates within membrane protrusions (Fig. 5). Collectively, these findings suggest that CD38 may participate in membrane microdomains involved in cell adhesion and motility *via* NAD^+^ independent interactions.

The partial overlap between the A549 and THP-1 CD38-proximal proteomes further prompted us to consider whether CD38 might contribute to TEM in tumor cells in a manner analogous to its reported functions in immune-cell extravasation (9). To explore this possibility, we examined A549 TEM using HUVEC-T1 monolayers. Rather than blocking the known CD38 ligand PECAM-1 with antibodies, we used soluble recombinant CD38 to occupy potential endothelial ligands in a hypothesis-free fashion. Although this strategy would be expected to provide weaker inhibition than direct antibody-mediated blockade of PECAM-1, it nevertheless produced a significant reduction in A549 transmigration. The relatively modest effect size (∼11%) suggests that CD38 is unlikely to act as a dominant adhesion receptor by itself. Instead, our results support a model in which CD38 contributes to TEM through participation in larger adhesive protein networks that warrant further investigation. Given the enrichment of proteins involved in ECM remodeling in both A549 and THP-1 cells, it is also possible that CD38 contributes to ECM invasion through regulation of cell–matrix interactions, although this possibility remains beyond the scope of the present study.

The migration phenotype and adhesion-associated proteomic signatures led us to hypothesize that CD38 may participate in protein networks formed at the tumor–endothelial interface. To investigate this possibility, we combined NBID with SILAC labeling to characterize cell-specific CD38-proximal proteomes in a tumor–endothelial coculture system. HUVEC-T1 cells were selected because they express high levels of PECAM-1 (Supplemental Fig. S2; Supplemental Table S11), the only experimentally validated CD38 ligand reported to date, while expressing no detectable CD38 under our experimental conditions. Based on this setup, NBID was expected to enrich CD38-proximal surface proteins from the A549 cell surface—as in the monoculture—and from intercellular complexes in tumor–endothelial junctions that incorporated A549 derived CD38. Compared with A549 monocultures, fewer proteins were identified in the coculture experiments, likely reflecting reduced tumor-cell input and complications introduced by the SILAC-DIA workflow. Nevertheless, several proteins, including EPHB2, DSG2, PLXNB2, and CD99, were enriched specifically in cocultured A549 cells. More importantly, cadherin family members CDH2 and DSG2 were enriched in both A549 and endothelial SILAC channels (Fig. 7). Because both proteins are capable of homophilic intercellular interactions (42, 44), these findings suggest that CD38 may be associated with cadherin-mediated adhesion structures spanning the tumor–endothelial interface. While canonical desmosomal adhesion typically involves both desmogleins and desmocollins (41), the absence of enrichment of desmocollins in our datasets suggests that the observed DSG2 signal may reflect homophilic adhesion complexes rather than canonical desmosomes.

Interestingly, despite the reported interaction between CD38 and PECAM-1 (9), PECAM-1 was not detected in either monoculture or coculture NBID datasets. Several explanations may account for this observation. The interaction may be absent under our experimental conditions, may occur too transiently, or may be insufficiently biotinylated by TurboID. It is also possible that binding of the targeting nanobody interfered with the interaction if the relevant binding interface overlaps with the MU1053 epitope. Because the molecular interface between CD38 and PECAM-1 remains unknown, we attempted structural prediction using AlphaFold3. Although some predicted models suggested potential overlap between the putative interface and the MU1053 epitope, the low confidence score of these models was insufficient to support meaningful conclusions (data not shown). Consequently, the contribution of CD38–PECAM-1 interactions to tumor–endothelial adhesion in our experimental system remains unresolved and warrants further investigation.

From a methodological perspective, NBID expands the growing repertoire of PL approaches targeting endogenous cell-surface proteins. The development of NBID was initially motivated by the practical limitations of antibody-based PL in large-scale adherent-cell cultures, where antibody consumption can become prohibitively expensive. Recent studies have reported related strategies using recombinant antibody–AirID and Fab–AirID conjugates to investigate EGFR-associated proteins under different stimulations (30). Although developed independently, these approaches and NBID share several conceptual advantages, including the use of purified affinity reagents to target endogenous proteins under cultured conditions. Earlier studies have also demonstrated the utility of nanobody-guided PL through genetically encoded GFP nanobody–enzyme fusion systems for tissue-specific labeling and intracellular protein-network analysis (45, 46). These studies provided important proof-of-principle support for nanobody-guided labeling, whereas NBID extends the concept to purified POI-specific NTCs that directly target endogenous surface proteins without genetic manipulation and affinity tags. The experimental workflow was further inspired by previously established antibody-based PL strategies, including ProtA–TurboID and LUX-MS (20, 21). Overall, NBID complements existing proximity-labeling methodologies while providing several practical advantages, including scalable production, simplified workflows, compatibility with native cell culture conditions, and reduced enzyme-to-target distance.

Despite these advantages, NBID shares several limitations with other affinity reagent-based PL approaches. Epitope occlusion—competition between nanobody binding and endogenous protein interactions—and restricted epitope accessibility within protein complexes may all influence labeling outcomes. Where possible, the use of nanobodies recognizing distinct epitopes may help evaluate such effects. Antibody or nanobody affinity and specificity also need to be validated, as exemplified in recent research using the BAR approach (47). In addition, background arising from nonspecific protein capture and endogenous biotinylation can limit sensitivity when studying the protein neighborhood of low-abundance proteins. Control selection also warrants consideration. Unlike conventional antibodies, nanobodies do not contain Fc domains that may introduce unwanted interactions. In the present study, we therefore used unconjugated MU1053 together with free TurboID as a control, maintaining comparable nanobody and enzyme concentrations while preventing target-directed labeling. This design is conceptually similar to the use of freely expressed enzyme controls in genetically encoded proximity-labeling experiments, and the addition of unconjugated MU1053 also allowed us to account for any potential effects arising from nanobody binding itself, although such effects are expected to be minimal. More broadly, control strategies vary substantially across affinity-based proximity-labeling platforms. For example, LUX-MS employs identical antibody–photocatalyst complexes in both experimental and control samples but omits photoactivation in the control condition (21), whereas an example of BAR studies have used controls lacking primary antibodies altogether (47). Because the labeling enzyme in NBID and other antibody-based approaches is supplied exogenously rather than continuously expressed throughout cell culture, background biotinylation is inherently reduced, providing flexibility in control design compared with genetically encoded approaches. Nevertheless, alternative control designs may be advantageous in applications where substantial nonspecific enrichment is observed.

In summary, we established a PL workflow based on POI-specific NTCs and applied it to characterize CD38-associated membrane protein networks in tumor and immune-cell models. Our results revealed enrichment of proteins involved in cell adhesion, ECM remodeling, and Wnt signaling, identified CD38-associated membrane protrusion structures, and supported a role for CD38 in TEM. By combining NBID with SILAC-based coculture proteomics, we further identified CD38-associated cadherin adhesion structures containing CDH2 and DSG2 at the tumor–endothelial interface. Together, these findings provide new insight into the membrane-associated functions of CD38 and establish NBID as a versatile platform for investigating endogenous surface-protein environments.

## Data Availability

The mass spectrometry proteomics data have been deposited to the ProteomeXchange Consortium *via* the MassIVE partner repository and are available *via* MassIVE identifiers MSV000100337, MSV000100338, and MSV000102176.

## Supplemental data

This article contains supplemental data.

## Conflict of Interest

The authors declare no competing interests.
