# Supplementary material for: Exploring the CD38-Associated Surfaceome via Nanobody-Targeted TurboID
Source: Mol Cell Proteomics. 2026 Jul 14;25(8):101623. doi: 10.1016/j.mcpro.2026.101623 (PMC13486792; doi:10.1016/j.mcpro.2026.101623)
Supplement: Supplementary file 1 [file mmc7.pdf]

supplemental file S7: A549 transendothelial migration quantification & associated flow cytometry report

Summary			
Sample ID	Treatment	CFDA-SE+ Events (60 s Acquisition)	
001-0_001	BSA	1690	
001-0_002	CD38	1486	
001-0_003	BSA	1781	
001-0_004	CD38	1584	
001-0_005	BSA	1826	
001-0_006	CD38	1645	
Treatment	Mean	SD	SEM
BSA	1765.6667	69.28443789	40.00139
CD38	1571.6667	80.21429632	46.31175
Ratio (CD38/BSA)		Student's t-test p	
0.890126487		0.033852356	

BD FACSDiva 8.0

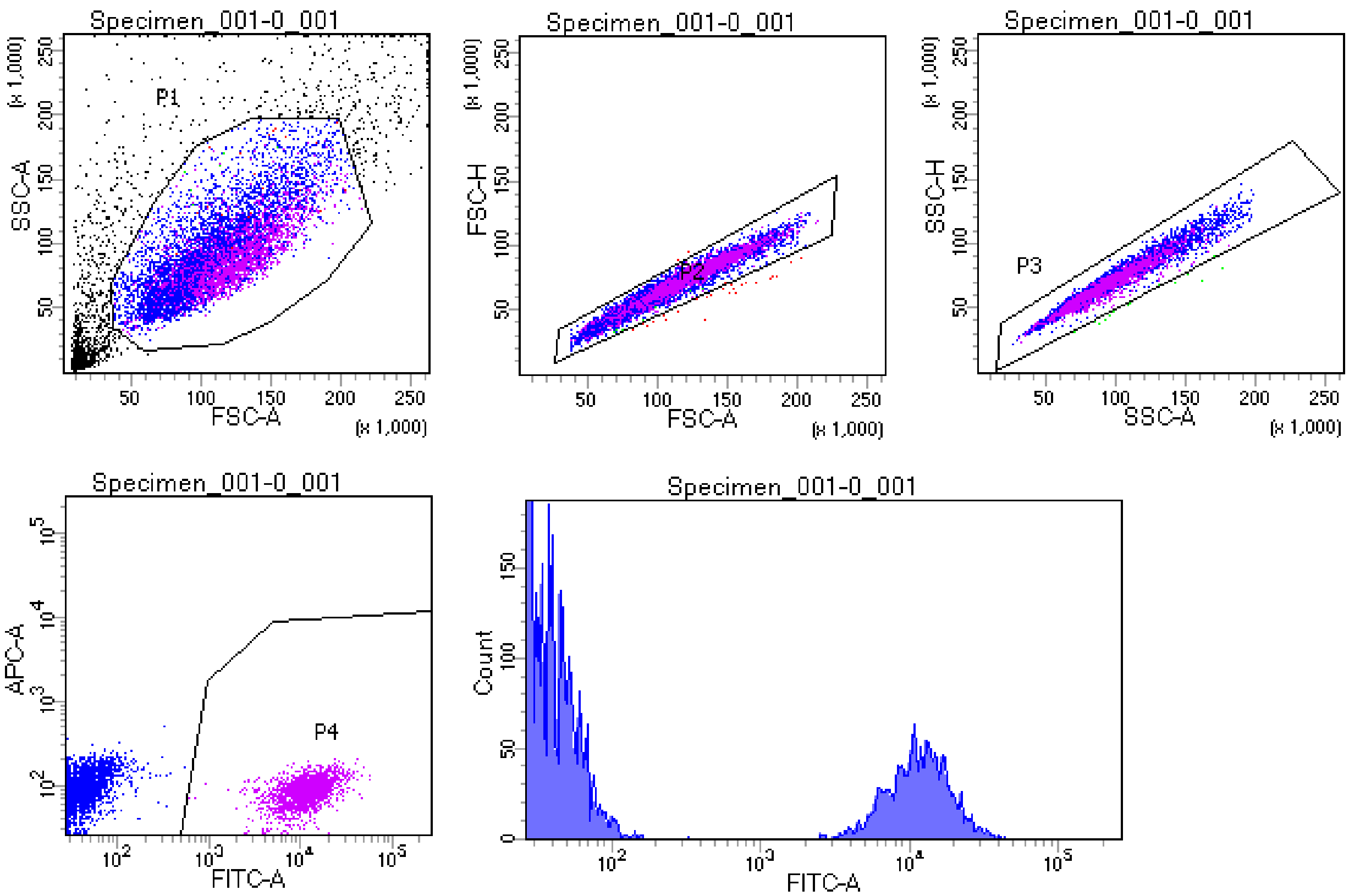

Tube: 0_001

Population	#Events	%Parent	%Total
All Events	9,912	####	100.0
P1	7,586	76.5	76.5
P2	7,556	99.6	76.2
P3	7,541	99.8	76.1
P4	1,690	22.4	17.1

Experiment Name:	20241101
Specimen Name:	Specimen_001
Record Date:	Nov 1, 2024 10:25:21 AM
$OP:	Administrator
GUID:	859d77c0-dd25-4d8b-a287-bac...
Tube Name:	0_001

Population	%Parent	FITC-A Mean	FITC-A Median
All Events	####	2,236	29
P1	76.5	2,789	32
P2	99.6	2,793	32
P3	99.8	2,785	32
P4	22.4	12,332	11,072

BD FACSDiva 8.0

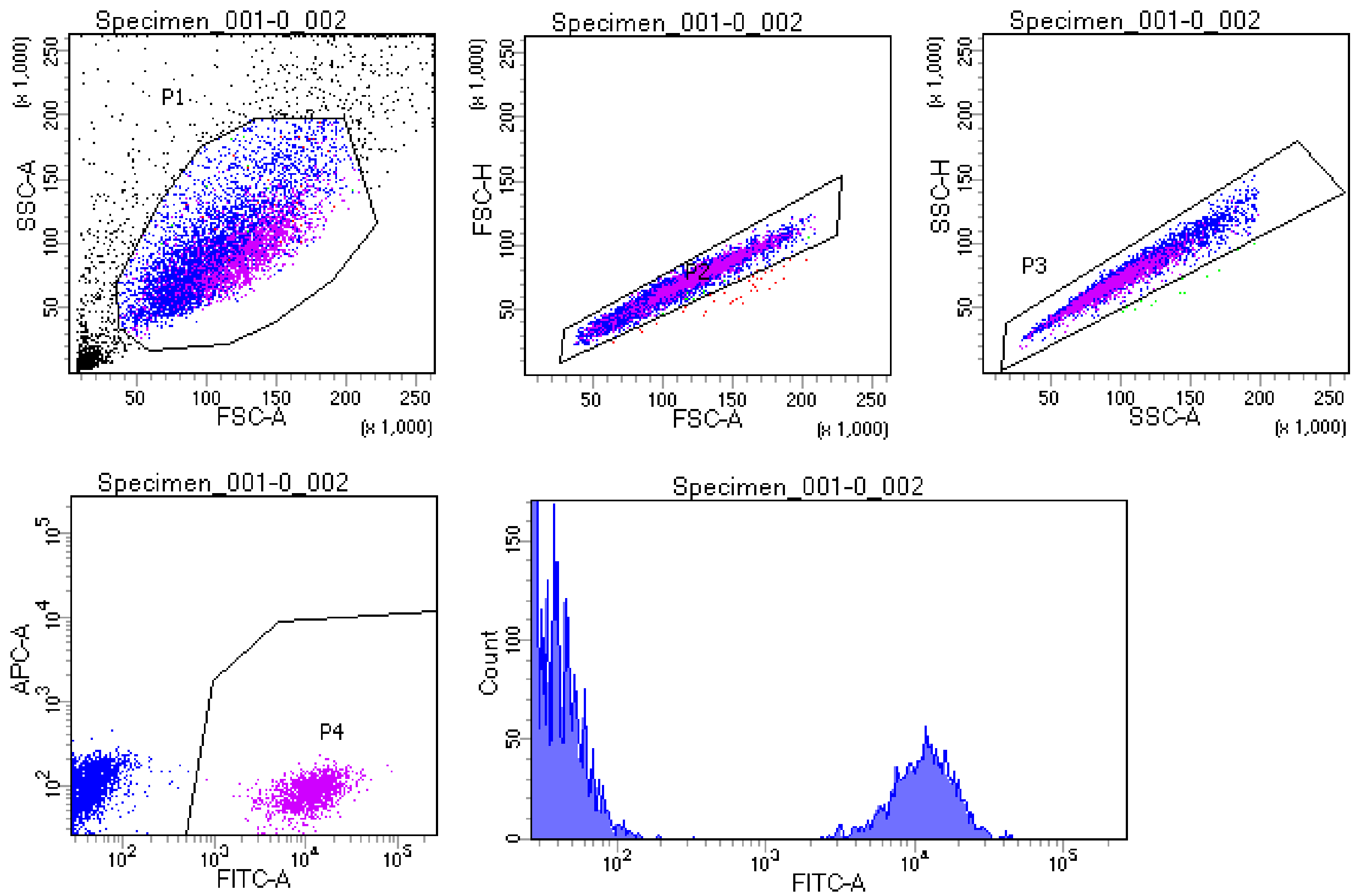

Tube: 0_002

Population	#Events	%Parent	%Total
All Events	8,935	####	100.0
P1	6,681	74.8	74.8
P2	6,637	99.3	74.3
P3	6,621	99.8	74.1
P4	1,486	22.4	16.6

Experiment Name:	20241101
Specimen Name:	Specimen_001
Record Date:	Nov 1, 2024 10:27:56 AM
$OP:	Administrator
GUID:	4adcda50-0638-41dd-91b0-aad...
Tube Name:	0_002

Population	%Parent	FITC-A Mean	FITC-A Median
All Events	####	2,205	27
P1	74.8	2,795	32
P2	99.3	2,790	32
P3	99.8	2,791	32
P4	22.4	12,341	11,300

BD FACSDiva 8.0

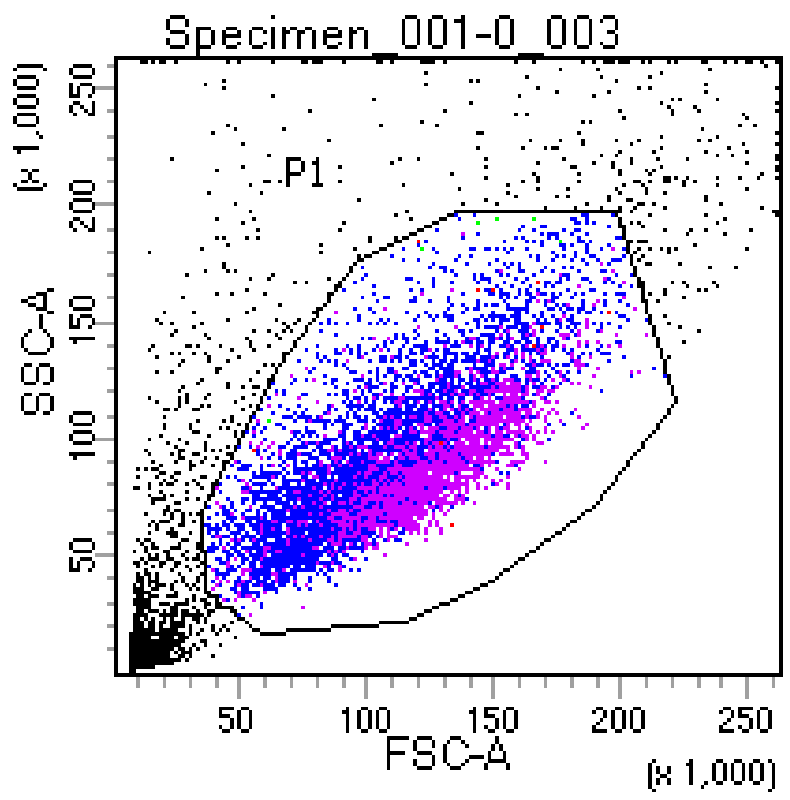

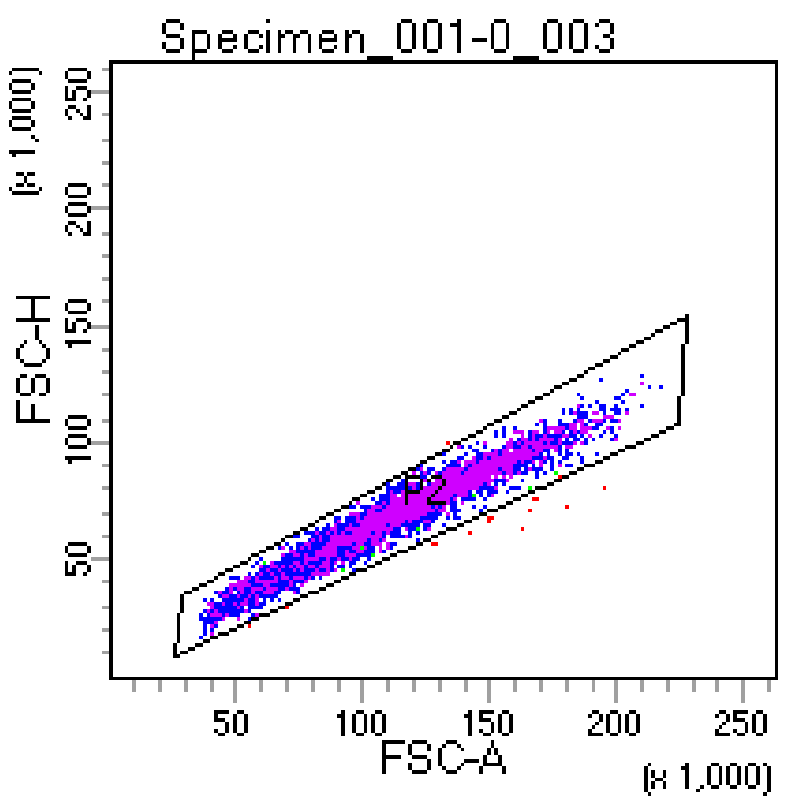

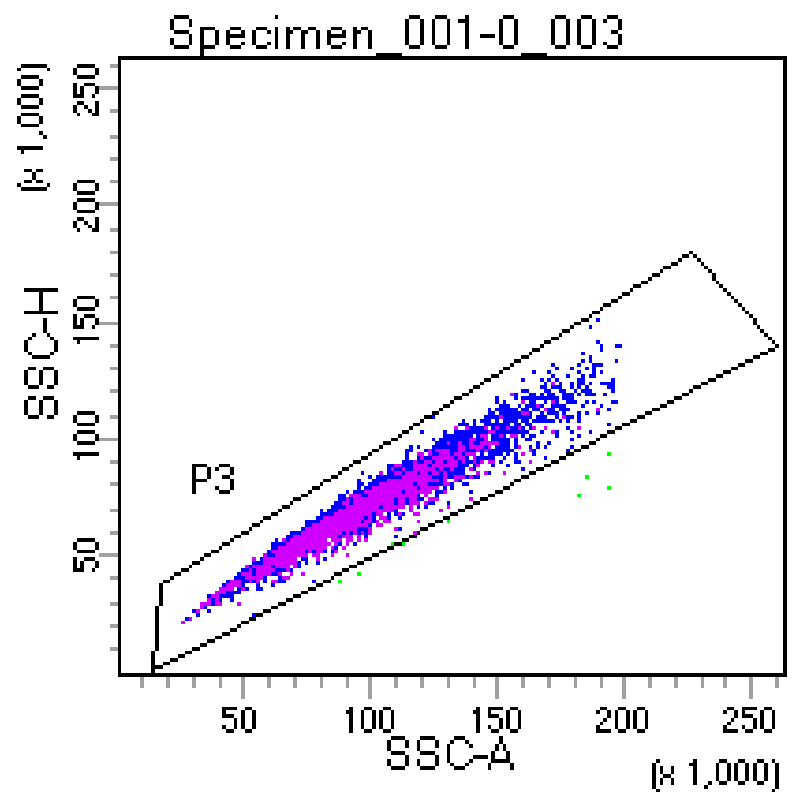

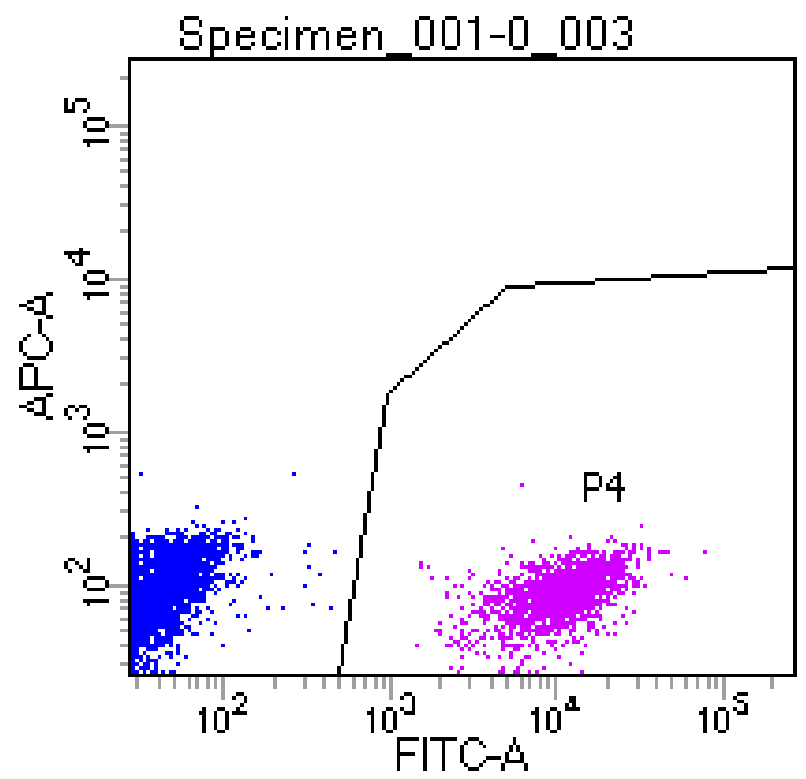

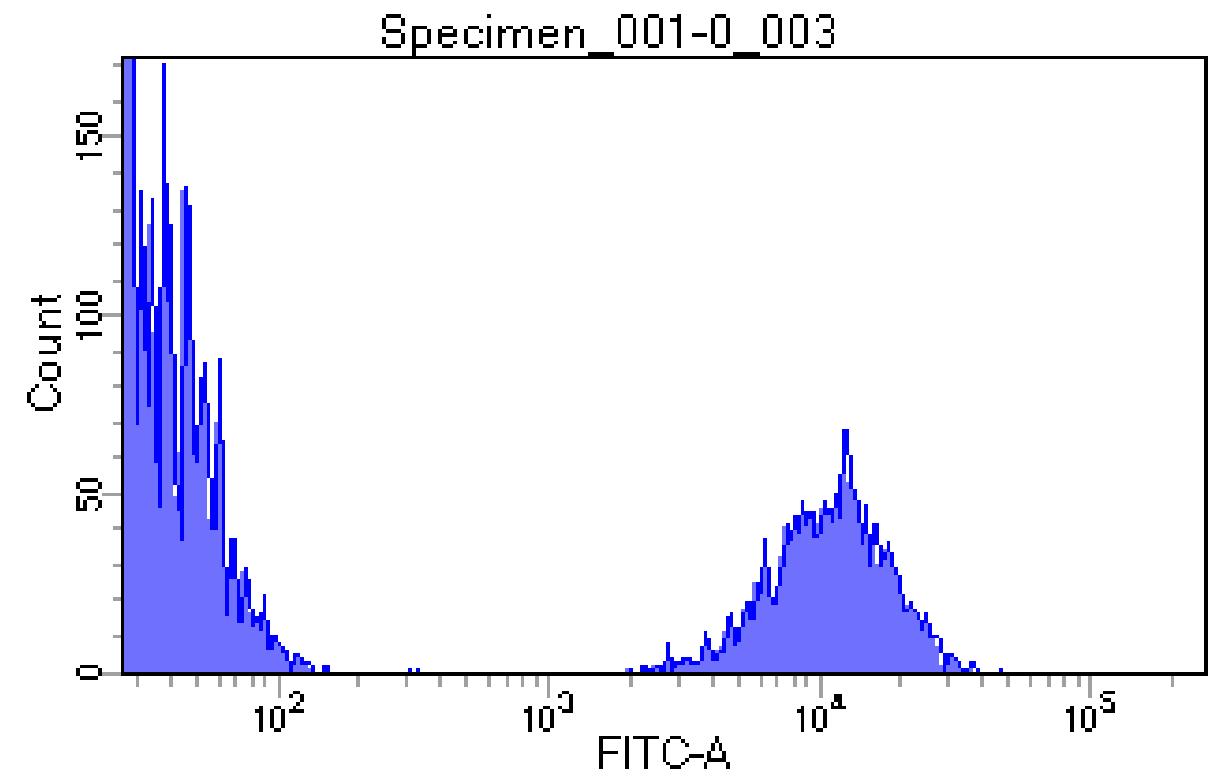

Tube: 0_003

Population	#Events	%Parent	%Total
All Events	9,846	####	100.0
P1	7,405	75.2	75.2
P2	7,386	99.7	75.0
P3	7,376	99.9	74.9
P4	1,781	24.1	18.1

Experiment Name:	20241101
Specimen Name:	Specimen_001
Record Date:	Nov 1, 2024 10:30:28 AM
$OP:	Administrator
GUID:	f95a1ce0-593b-4240-afff-4639a...
Tube Name:	0_003

Population	%Parent	FITC-A Mean	FITC-A Median
All Events	####	2,225	27
P1	75.2	2,829	32
P2	99.7	2,832	32
P3	99.9	2,831	32
P4	24.1	11,642	10,552

BD FACSDiva 8.0

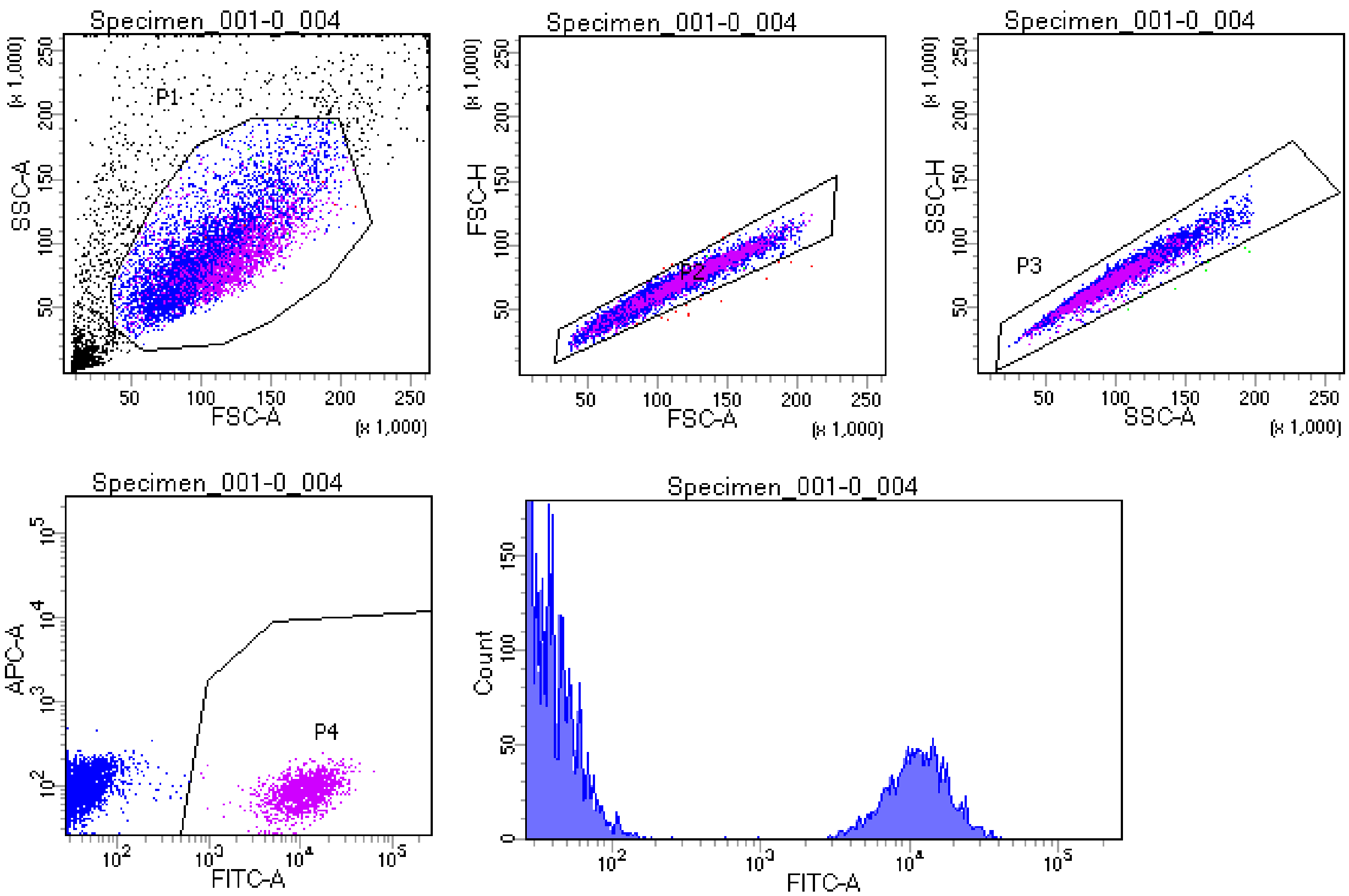

Tube: 0_004

Population	#Events	%Parent	%Total
All Events	9,800	####	100.0
P1	7,346	75.0	75.0
P2	7,324	99.7	74.7
P3	7,316	99.9	74.7
P4	1,584	21.7	16.2

Experiment Name:	20241101
Specimen Name:	Specimen_001
Record Date:	Nov 1, 2024 10:33:04 AM
$OP:	Administrator
GUID:	e8e4f64c-fb5b-41d1-a48a-1e04...
Tube Name:	0_004

Population	%Parent	FITC-A Mean	FITC-A Median
All Events	####	2,088	28
P1	75.0	2,640	31
P2	99.7	2,640	31
P3	99.9	2,635	31
P4	21.7	12,072	10,942

BD FACSDiva 8.0

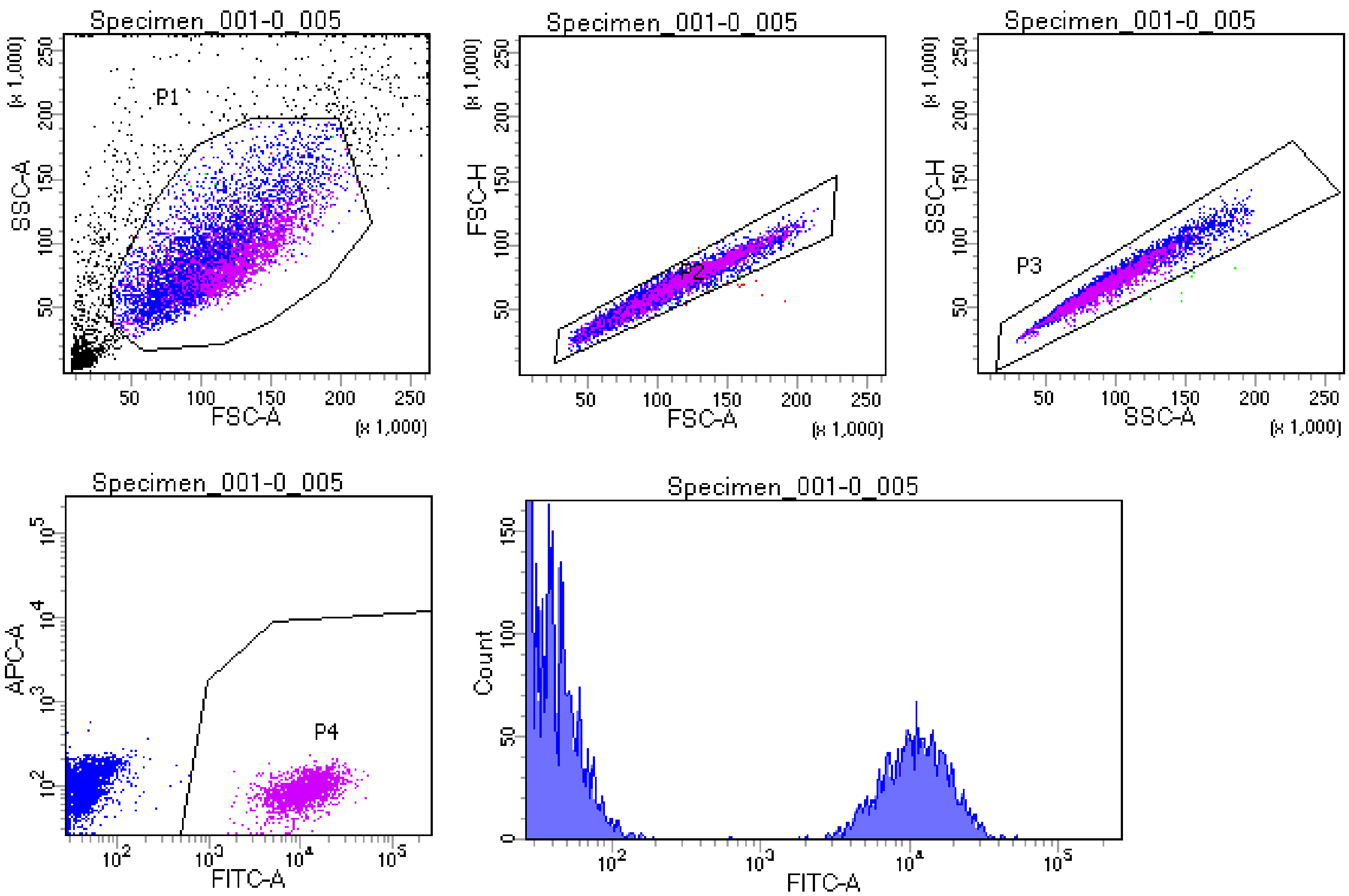

Tube: 0_005

Population	#Events	%Parent	%Total
All Events	9,401	####	100.0
P1	7,042	74.9	74.9
P2	7,025	99.8	74.7
P3	7,012	99.8	74.6
P4	1,826	26.0	19.4

Experiment Name:	20241101
Specimen Name:	Specimen_001
Record Date:	Nov 1, 2024 10:35:33 AM
$OP:	Administrator
GUID:	18d2a674-6c4b-43c1-b647-844...
Tube Name:	0_005

Population	%Parent	FITC-A Mean	FITC-A Median
All Events	####	2,492	30
P1	74.9	3,143	34
P2	99.8	3,146	34
P3	99.8	3,139	34
P4	26.0	11,975	10,679

BD FACSDiva 8.0

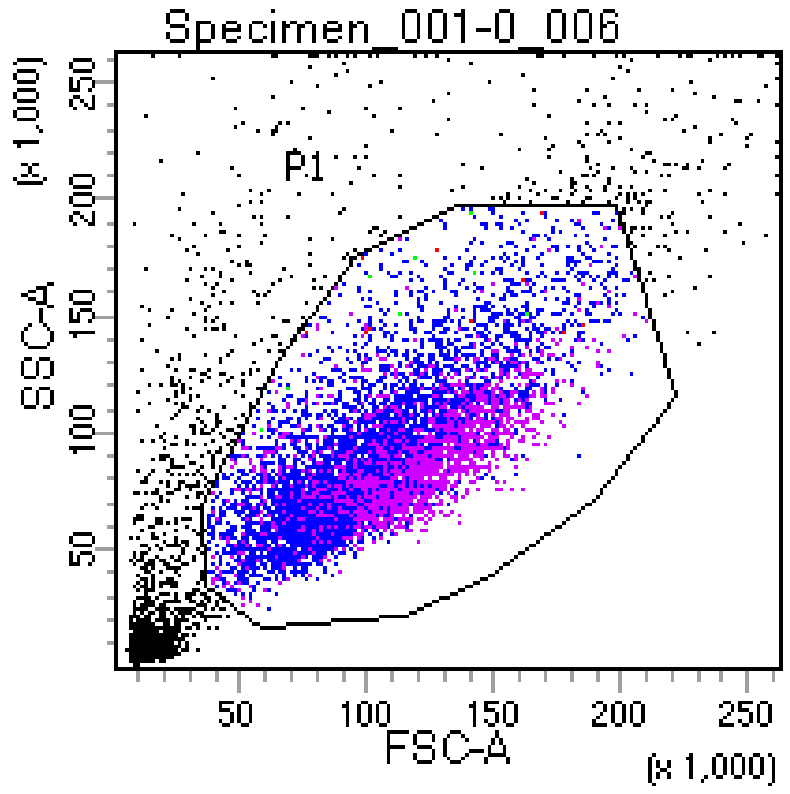

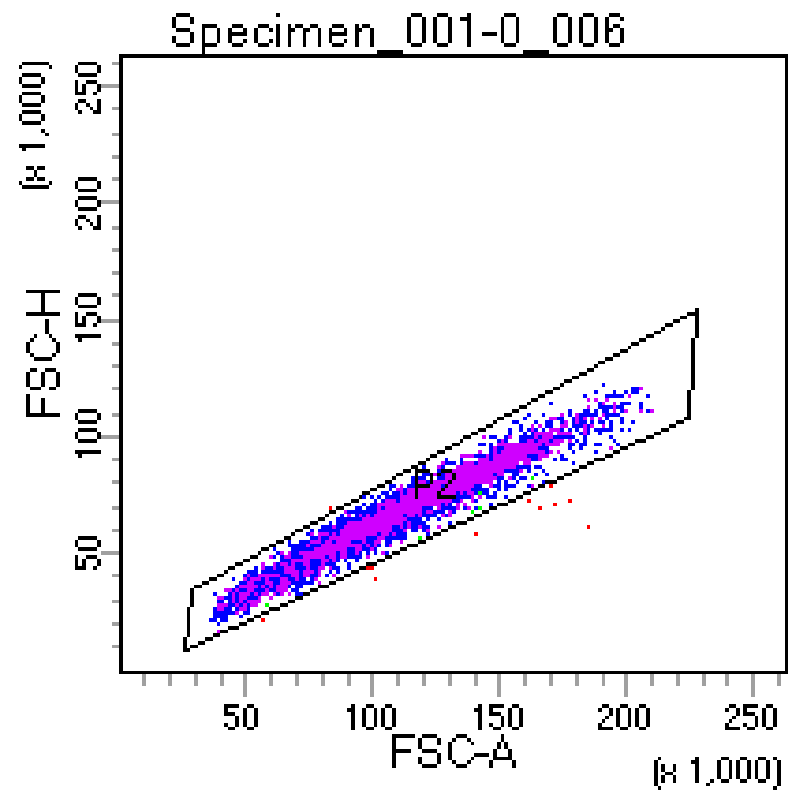

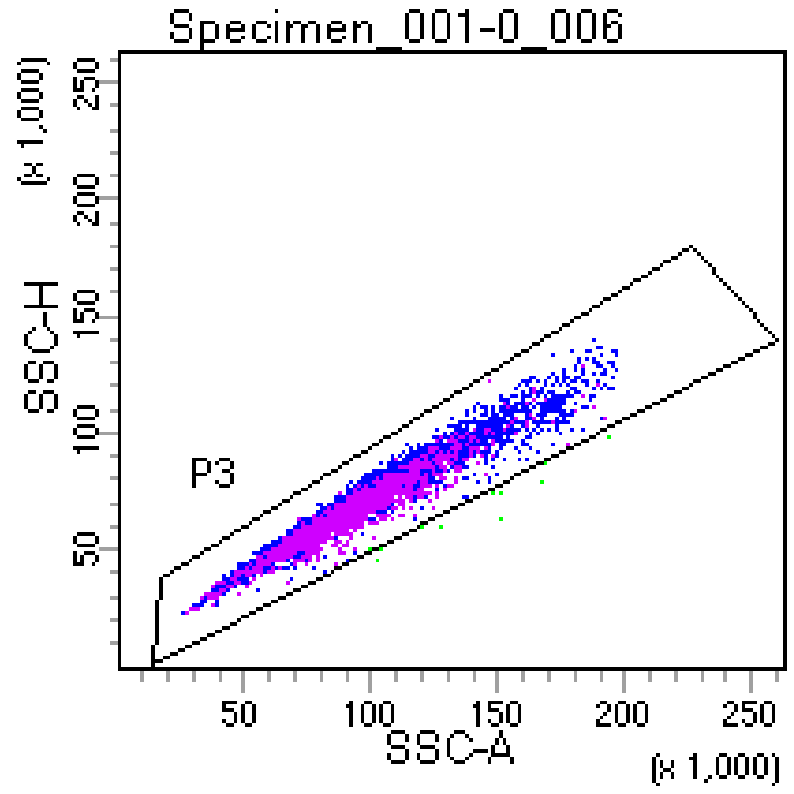

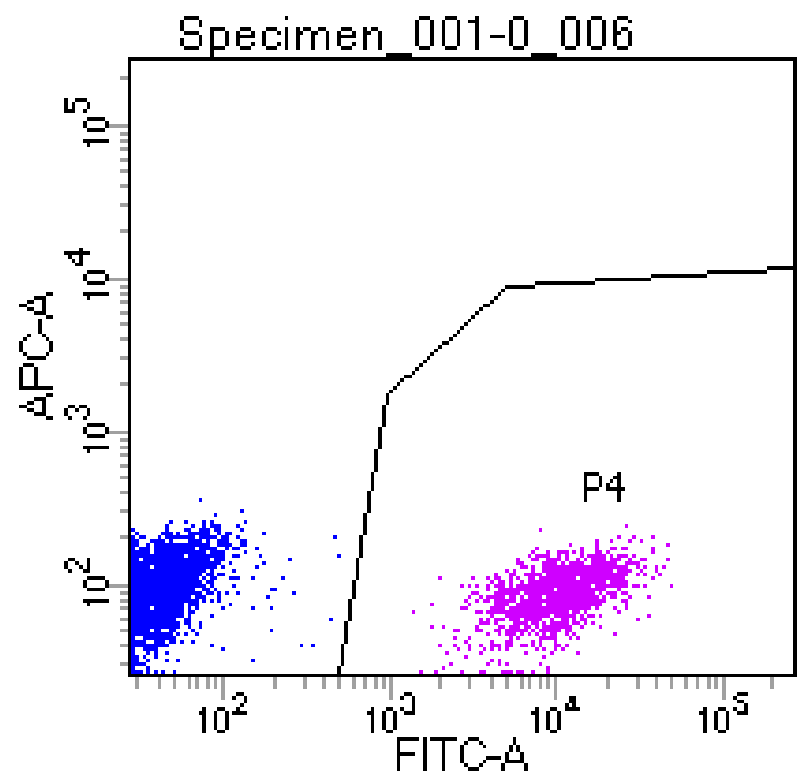

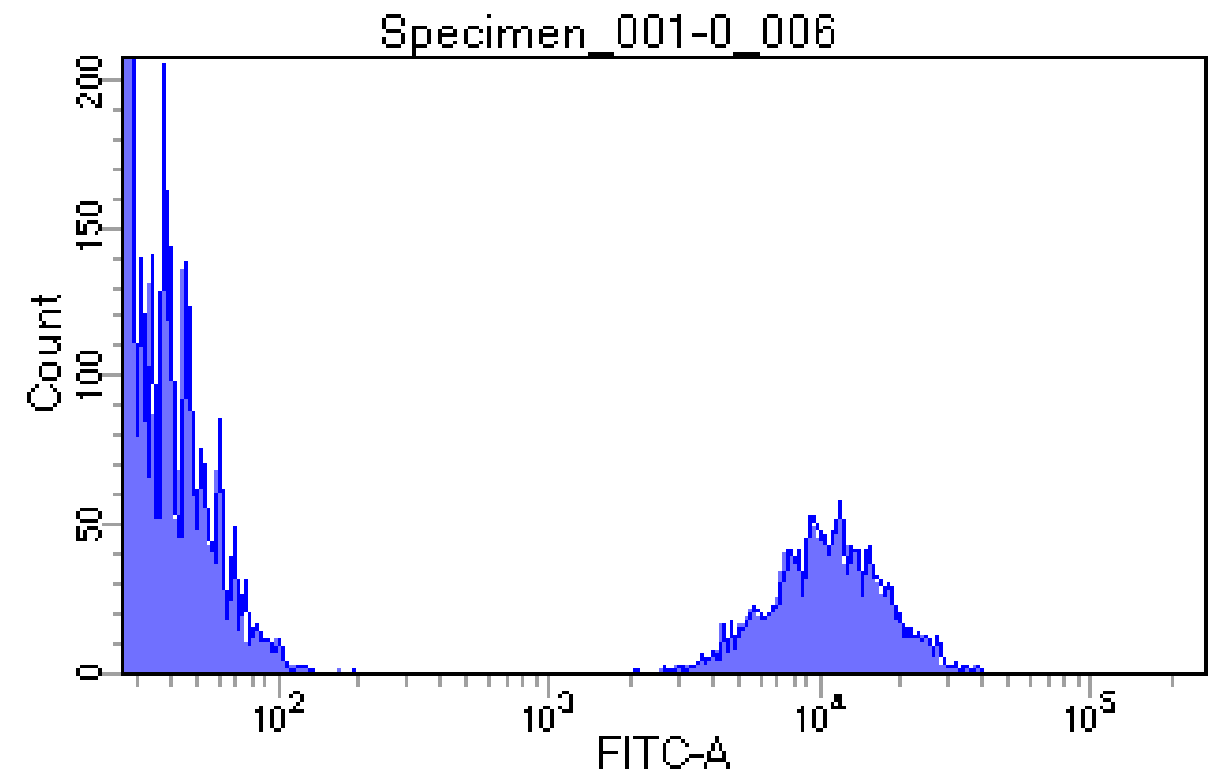

Tube: 0_006

Population	#Events	%Parent	%Total
All Events	10,002	####	100.0
P1	7,288	72.9	72.9
P2	7,271	99.8	72.7
P3	7,259	99.8	72.6
P4	1,645	22.7	16.4

Experiment Name:	20241101
Specimen Name:	Specimen_001
Record Date:	Nov 1, 2024 10:38:08 AM
$OP:	Administrator
GUID:	c734c604-c993-4457-9d9a-0be...
Tube Name:	0_006

Population	%Parent	FITC-A Mean	FITC-A Median
All Events	####	2,058	27
P1	72.9	2,657	31
P2	99.8	2,656	31
P3	99.8	2,644	31
P4	22.7	11,580	10,320
